# Molecular Profiles of Pre- and Postoperative Breast Cancer Tumours Reveal Differentially Expressed Genes

**DOI:** 10.5402/2012/450267

**Published:** 2012-11-26

**Authors:** Margit L. H. Riis, Torben Lüders, Elke K. Markert, Vilde D. Haakensen, Anne-Jorun Nesbakken, Vessela N. Kristensen, Ida R. K. Bukholm

**Affiliations:** ^1^Department of Surgery, Akershus University Hospital, 1478 Lørenskog, Norway; ^2^Institute for Clinical Medicine, Faculty of Medicine, University of Oslo, 0318 Oslo, Norway; ^3^Department of Clinical Molecular Biology and Laboratory Sciences (EpiGen), Akershus University Hospital, 1478 Lørenskog, Norway; ^4^The Simons Center for Systems Biology, Institute for Advanced Study, Princeton, NJ 08540, USA; ^5^Department of Genetics, Institute for Cancer Research, Oslo University Hospital Radiumhospitalet, 0310 Oslo, Norway; ^6^Department of Pathology, Akershus University Hospital, 1478 Lørenskog, Norway; ^7^Institute of Health Promotion, Akershus University Hospital, 1478 Lørenskog, Norway

## Abstract

Gene expression studies on breast cancer have generally been performed on tissue obtained at the time of surgery. In this study, we have compared the gene expression profiles in preoperative tissue (core needle biopsies) while tumor is still in its normal milieu to postoperative tissue from the same tumor obtained during surgery. Thirteen patients were included of which eleven had undergone sentinel node diagnosis procedure before operation. Microarray gene expression analysis was performed using total RNA from all the samples. Paired significance analysis of microarrays revealed 228 differently expressed genes, including several early response stress-related genes such as members of the *fos* and *jun* families as well as genes of which the expression has previously been associated with cancer. The expression profiles found in the analyses of breast cancer tissue must be evaluated with caution. Different profiles may simply be the result of differences in the surgical trauma and timing of when samples are taken and not necessarily associated with tumor biology.

## 1. Introduction

Breast cancer is detected either by clinical signs such as palpable tumour or in mammographic screening. In both cases biopsies are taken from the tumour to determine whether the tumour is benign or malign. If malignancy is detected, the patient will be scheduled for surgery within a few weeks. Before the surgery, sentinel node (SN) diagnostics is generally performed to examine the spread of cancer cells to axillary lymph nodes. The SN can be identified using a blue dye, a radioactive colloid, or a combination of the two [1, 2].

Microarray technology enables scientists to study thousands of genes simultaneously. The resulting molecular profile can be used to study complex multifactorial diseases such as breast cancer [3, 4]. Gene signatures have been shown to correlate with clinically relevant clinicopathological parameters and prognosis [5–7]. These molecular signatures may be used to predict the individuals for whom therapy is beneficial and spare unnecessary treatment for over 80% of the others [6, 8–10].

The time of procurement, which refers to the point of when the biopsies are taken [11] as well as the postoperative handling [12], has been found to be a confounding factors in microarray data analysis in breast cancer. Most of the previously published studies consist of tumour tissue taken in connection to surgery. Biopsies taken from the tumour, while the tumour is within the breast prior to any manipulation, must be as near to the true expression state as possible. In this study, we analyzed whether there are differences in genes expressed in preoperative biopsies obtained in connection with mammography and postoperative biopsies taken from the tumour immediately after its removal from the patient.

## 2. Materials and Methods

### 2.1. Patients

This study includes 13 patients from which both a pre- and postoperative samples were available. Histopathological characteristics are listed in Table 1. All of the patients had been operated with ablatio mammae (surgical removal of the entire breast). Sentinel node diagnostics using 99 m Tc-labelled colloids were performed in eleven patients as a part of the surgical procedure [13], while the remaining two underwent direct complete axillary dissection. No recurrence of disease has been observed so far for these patients, but the follow-up time is short. All women participating in this study have signed an informed consent and the study design is approved by the Regional Committee for Medical and Health Research Ethics (REK).

### 2.2. Tissue Collection

Preoperative needle biopsies were obtained by an experienced radiologist using a 16 Gauge core needle device through a small skin incision in a sterile field. Three samples were processed for routine histological diagnosis while one sample for molecular analysis was put directly into RNAlater (Sigma Aldrich, St Louis, MO, USA). The postoperative samples were taken by the breast cancer surgeon upon removal of the breast and were preserved in RNAlater. The RNAlater-stabilised tissue samples were stored at −80°C. The time delay between the sampling of the pre- and postoperative specimens were 2–8 weeks.

### 2.3. RNA Isolation

RNA was prepared using the method of Wei and Khan [14] but modified to also include miRNA. Briefly, frozen tissue samples were homogenized in TriReagent (Ambion, Austin, TX, USA) using a 5 mm steal bead in a Mixer Mill MM301 (Retsch, Haan, Germany) at 30 Hz for 2 min. After phase separation with 0.2 vol chloroform, the aqueous phase (containing RNA) was mixed with 1.5 vol 100% ethanol and transferred to RNeasy Mini columns (Qiagen, Hilden, Germany). Further processing (including on-column DNase digestion) was per the manufacturer's protocol and the purified RNA was eluted with RNase-free water. RNA concentration was measured using NanoDrop ND-1000 UV-VIS Spectrophotometer (Thermo Fisher Scientific, Waltham, MA, USA) and the RNA quality analyzed on a 2100 Bioanalyzer (Agilent, Santa Clara, CA, USA). The purified RNA was stored at −80°C.

### 2.4. Microarray Analysis

mRNA amplification, labelling, and hybridization were done following the manufacturer's instructions (Agilent One-Color Microarray-Based Gene Expression Analysis; Version 5.7). Briefly, 500 ng RNA was amplified and labelled with Cy3 using the Quick Amp labelling kit and the labelled cRNA purified using the Qiagen RNeasy Mini Kit. Amplification and labelling efficiency were controlled on the NanoDrop before 1.65 *μ*g cRNA was fragmented and applied to Agilent Whole Human Genome 4 × 44 k microarrays (G4112F). After hybridisation for 17 h at 65°C the microarray slides were washed and scanned with the Agilent Microarray Scanner. Microarray data were extracted using Agilent Feature Extraction (v. 10.7.1.1) and further quantile normalized and analyzed using J-Express 2009 [15]. For expression values the gProcessedSignal from Feature Extraction were used and controls and bad spots were filtered with maximum 20% allowed missing values. The expression values were log2-transformed and missing values imputed using the LSImpute Adaptive method. Differential expression was analyzed using SAM as implemented in J-Express with 1000 permutations and only genes with false discovery rate (FDR) < 2.5% were considered significant. The microarray data are available at the ArrayExpress Archive (http://www.ebi.ac.uk/microarray-as/ae/) accession number E-MTAB-470.

Gene functional classification of the significant genes was performed using DAVID [16, 17] and pathway analysis was done through the use of Ingenuity Pathways Analysis (IPA; Ingenuity Systems, Redwood City, CA, USA).

### 2.5. Quantitative RT-PCR

To confirm the results of the microarray experiment, qRT-PCR using TaqMan low density arrays (Applied Biosystems, Carlsbad, CA, USA) were performed using primer-probe pairs for 13 of the significant genes (Table 2). The genes were selected to contain both up- and downregulated genes. 500 ng RNA was reverse transcribed using the High Capacity cDNA Reverse Transcription Kit (Applied Biosystems) per the manufacturer's instructions. Due to lack of material, qRT-PCR was only performed for ten of the sample pairs. The samples were further processed using TaqMan Gene Expression Master Mix (Applied Biosystems) and run on the 7900HT Real-Time PCR System (Applied Biosystems) as per the manufacturer's instructions. Relative changes in gene expression were analyzed using the ΔΔCt-method [18] with the preoperative sample as control sample for each pair. As endogenous controls the average of *GAPDH*, *18S*, and *ACTB* were used.

## 3. Results

### 3.1. mRNA Expression

**Figure 1:**
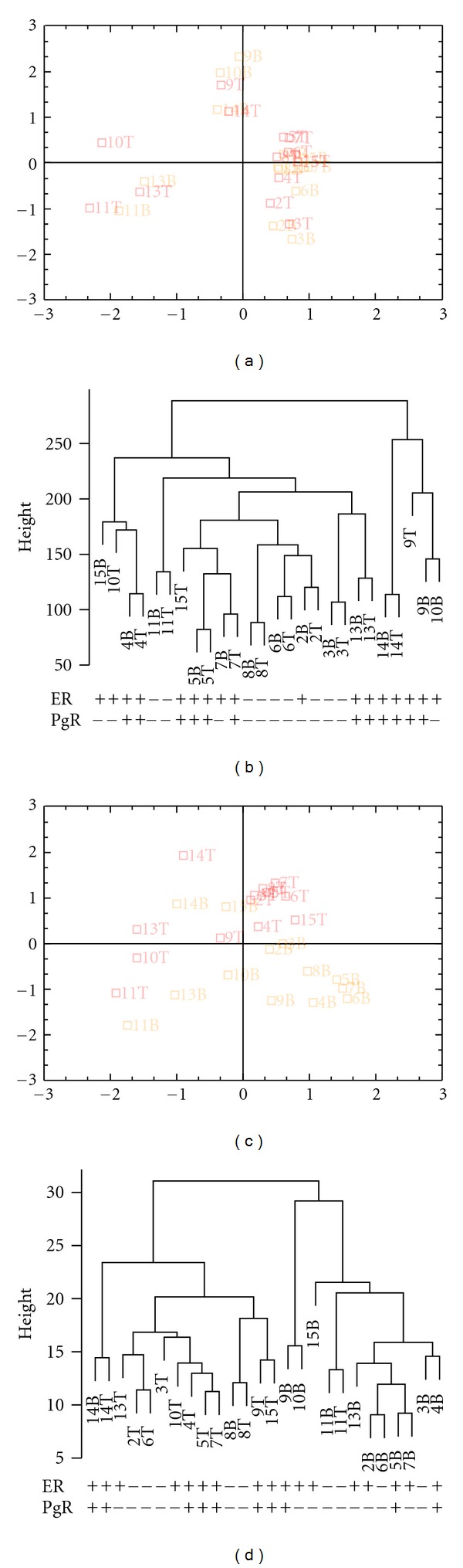
Unsupervised (a) and supervised (c) CA plots of mRNA expression in pre- and postoperative breast cancer tumours, and their corresponding clustering maps (b, d). The samples are marked by their respective numbers followed by either B, which defines the preoperative samples, or T, which defines the postoperative samples. The unsupervised chart was made using all 18,189 genes (24,105 probes) expressed on the microarrayss whereas for the supervised only the 228 genes (235 probes) with FDR < 2.5% from paired SAM were used.

RNA was isolated from matching samples taken both before and after breast cancer surgery of 13 patients. After filtering, expression data were available from 24,105 different probes representing 18,189 different genes. Comparing the gene expressions of the 13 pairs showed that there was relatively little difference between the pre- and postoperative samples (Figures 1(a) and 1(b) and Supplementary Figure  1 available online at doi:10.5402/2012/450267). Paired significance analysis of microarrays (SAM) [19], however, showed differently expression for 235 probes with false discovery rate (FDR) <2.5%, corresponding to 228 different genes (Supplementary Table  1) that separates the pre- and postoperative samples (Figures 1(c) and 1(d) and Supplementary Figure  2). The majority (201) of these genes were upregulated and only 27 were downregulated in the postoperative samples. The differentially expressed genes contained genes involved in early response such as* FOSB*, response to oxidative stress such as *DUSP1,9* as well as genes earlier identified as differentially expressed in cancer (*MAPK*,* MALAT1*,* RASD1*, etc) (Table 3).

**Figure 2:**
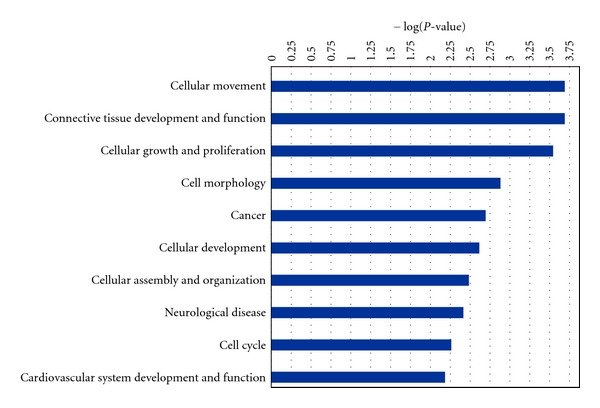
The most significantly enriched biological categories as identified with Ingenuity Pathway Analysis. For each category −log(*P* value) is reported.

**Figure 3:**
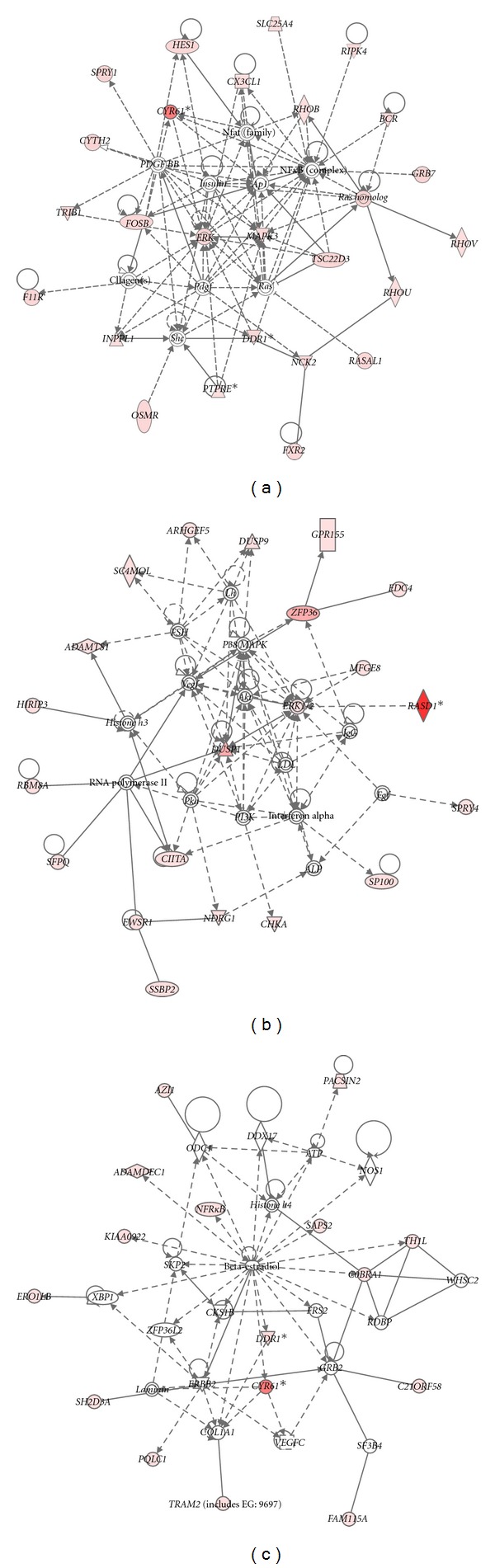
Most enriched molecular networks showing interactions between the significant genes (according to SAM)—(a) *FOSB*, *ERK*, *MAPK3*, *CYR61*, and the *RAS*-genes; (b) *DUSP1*, *ERK1/2*, *P38MAPK*, *DUSP9*, and *RASD1*; (c) *CYR61* and *NFR*κ*B*. The gene identifiers and corresponding expression values were uploaded into in the Ingenuity Pathway Analysis. Networks were then algorithmically generated based on their connectivity in Ingenuity's Knowledge Base. Molecules are represented as nodes, and the biological relationship between two nodes is represented as an edge (line). The intensity of the node colour indicates the degree of (red) up- or (green) downregulation. Nodes are displayed using various shapes that represent the functional class of the gene product.

Gene functional classification in DAVID of the upregulated genes showed enrichment for four groups (kinase/phosphatase, Ras, negative regulation of transcription, and transmembrane) while the downregulated genes mainly correspond to transmembrane proteins (Table 4). Gene function was also analyzed by Ingenuity Pathways Analysis (IPA) and includes “cellular movements,” “connective tissue development and movement” and “cellular growth and proliferation” (Figure 2). IPA also identified molecular networks connecting several of the genes: *FOSB*, *ERK*, *MAPK3*, *CYR61*, and the *RAS*-genes (Figure 3(a)); *DUSP1*, *ERK1/2*, *P38MAPK*, *DUSP9*, and *RASD1* (Figure 3(b)); *CYR61* and *NFR*κ*B* (Figure 3(c)) amongst other (Supplementary Figure  3).

### 3.2. Quantitative RT- PCR Validation

**Figure 4:**
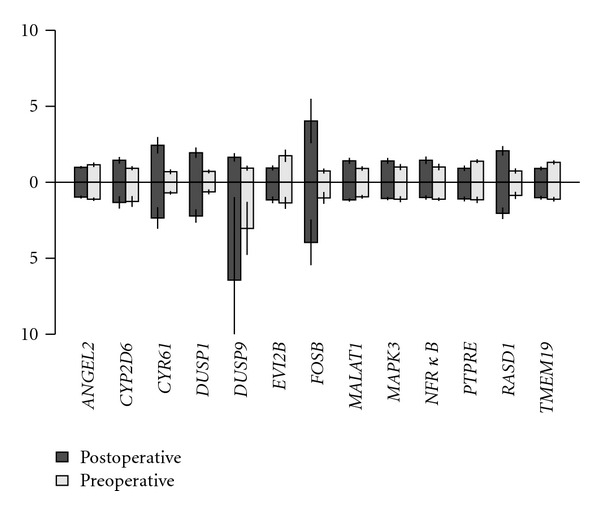
Relative expression of selected genes on microarrays (upwards) and qRT-PCR (downwards). Values shown are mean expression ±SE. For easier comparison, both the microarray and qRT-PCR values were gene-centered.

To confirm the results of the microarray experiment, qRT-PCR was performed using primer-probe pairs for the top significant genes. The genes were selected to contain both up- and downregulated genes. The microarray and the qRT-PCR results were in agreement with the following genes (Figure 4 and Table 2): *ACTB, CYR61, DUSP1, EVI2b, FOSB, GAPDH*, and *RASD1*.

### 3.3. Histological Analysis versus Gene Expression Analysis

Immunohistochemistry was performed on the pre- and the postoperative samples. Overall the tumour content in the two samples were comparable and there was no systematic bias (Table 1), indicating that the gene expression as measured by microarray is comparable in the pairs. In addition, ER and PGR status for the pre- and postoperative specimen were similar (Table 1).

## 4. Discussion

Microarray studies have influenced breast cancer research over the last decade revealing breast cancer as a heterogeneous disease opening for individual treatment in a clinical perspective. Therefore, the results from microarray studies need to be validated. Multiple studies have generated different gene list and studied the reproducibility and correlation with prognosis [20–22]. Despite the difference in development of these signatures and the limited overlap in gene identity, they show similar prognostic performance, adding to the growing evidence that these prognostic signatures are of clinical importance [20]. There are two prospective ongoing studies, the MINDACT trial [23] in Europe and TAILORx [24] in USA which will evaluate the prognostic potential of this technology.

One important question may be if the differences in gene expressions are related to tumour biology or reflect the surgical trauma of the patient or the manipulation of the tumour tissue during the operative procedure or the time of specimen handoff. If altered gene expression is caused by such exogenous factors, the results may differ considerably between studies depending on the operative procedure and the time spent at the operation before taking the tissue samples. It is therefore important to evaluate if gene expression patterns differ between biopsies taken before and after surgical procedure. This has been done in our study with 13 patients and the gene list of 228 genes was dominated by stress-related genes like *CYR61*, *MALAT1*, *RASD1*, *CX3CL1*, *FOSB*, and *CYP2D6*. Some of these genes have been studied by others in relation to oxidative stress [25–27] and also psychological stress [28]. These genes have different functions all included in very important pathways with strong hubs such as *MAPK3*, *NFR*κ*B*, *FOS,* and *ERK*.

Upregulation of Fos has been associated with breast cancer in a number of studies [29–31]. The fos-gene family consists of 4 members: *FOS*, *FOSB*, *FOSL1*, and *FOSL2*. These genes encode leucine zipper proteins that can dimerise with proteins of the jun-family, and the Fos-proteins have been implicated as regulators of cell proliferation, differentiation, and transformation. Another gene, *CYR61* (cysteine-rich, angiogenic inducer, 61), most strongly associated to differential expression in pre- and postoperative samples, belongs to the CCN-family [32] and mediates cell proliferation, survival, and apoptosis. Acting as an extracellular matrix-associated signalling molecule, *CYR61* promotes the adhesion of endothelial cells through interaction with the integrin *α*v*β*3 and augments growth factor-induced DNA synthesis in the same cell type [33]. In this aspect, it is both chemotactic and angiogenic, two properties important for tumour growth and vascularisation. *CYR61* is claimed to play a critical role in oestrogen, as well as growth factor-dependent breast tumour growth [34]. In our list of genes, *CYR61* is repeatedly connected in most of the involved pathways. Further studies will be necessary to confirm and explain this association.

It is of particular importance to take into consideration knowledge about gene expression differences in pre- and postoperative tissue samples in the case of treatment response studies in the neoadjuvant setting, when the first sample is frequently taken by biopsy and the second during operation. In a study comparing gene expression profiles before and after doxorubicin and cyclophosphamide neoadjuvant chemotherapy [35] one of the genes upregulated after the first chemotherapy treatment was *DUSP1*. Expression of this gene may be associated with resistance to further administration of chemotherapy. In our study *DUSP1* was one of the significantly upregulated genes in postoperative tissue. *DUSP1* is a stress response gene of the mitogen-activated protein (*MAP*) kinase phosphatase family and is located in the cytoplasm, mitochondria, and the nucleus. The gene has been shown to be overexpressed in human breast cancer [36] through different signalling pathways. One important pathway is in response to stress which is mediated in part through the p38 *MAPK* pathway. Later studies have implicated that *DUSP1* is controlled by p53 during cellular response to oxidative stress [37]. A similar discussion could be relevant on molecular profiling of inflammatory breast cancer [29], where *DUSP1* was also among the genes suggested to be useful diagnostic and prognostic markers. Our study suggests that such findings have to (1) demonstrate upregulation above the one observed here by us attributable to the pre- and postoperative factors and (2) that deregulation attributable to the pre- and postoperative factors is similar in the compared case/control or treatment arm groups.

In the present study, it is not possible to separate the effects of operative manipulation, anaesthesia, or the injection of radioactive substance to examine spread of cancer cells. For the latter, we should have had a biopsy after the application of radioactivity not before operation. However, both ethical and logistical considerations make collection of such a sample infeasible. Wong et al. [11] studied the effects of timing of fine-needle aspiration biopsies. Using hierarchical clustering analysis, they found 12 genes to be differentially expressed before and after surgery, which were in agreement with our study all fos-related. However, it was unclear whether any other treatment, like sentinel node, was given to patients between the two time points. It has been previously shown that both fine-needle aspiration biopsy and central core biopsy yield a similar quality and quantity of total RNA and that microarray profiles are mainly the same [38]. Microscopic cell counts have demonstrated that there are more stromal cells present in core biopsies compared to fine-needle biopsies [38], and the core biopsy is therefore needed for the complete histological examination.

Another confounding factor in the analysis of gene expression profiles of breast cancers is intratumour heterogeneity [39, 40]. Even though this study was not designed to analyze this, molecular subclassification [41] of the samples did in a few cases give different result for the pre- and postoperative samples (Table 5). Interference from surrounding normal tissue is not likely since the overall gene expression profiles of the pre- and postoperative samples were very similar and distinct from that of adjacent normal tissue (Supplementary Figure 1), thus, suggesting true cases of intratumour heterogeneity.

As we have seen there are genes that are differently expressed between the pre- and postoperative samples. We compared our gene lists to some of the publically available gene list to see if there were any overlapping genes. The Oncotype DX consists of 21 genes, 16 cancer related genes, and 5 reference genes [42]. One of these is also found in our gene list which differs between pre- and postoperative samples, namely, *GRB7*, which is upregulated in the postoperative samples. *GRB7* was associated with an increased risk of recurrence in TNBC (tripple negative breast cancer) treated with adjuvant doxorubicin-containing chemotherapy, suggesting that *GRB7* or *GRB7*-dependent pathways may serve as potential biomarkers for therapeutic targets [43, 44]. We have shown that this gene is upregulated in the postoperative sample. Even though the gene has been well characterized *in vitro* [45–47], it is of interest that we find it in the list of genes separating pre- and postoperative samples.

We also wanted to compare the 70 genes listed in the Mammaprint which were based on the intrinsic gene list [6]. As with the Oncotype DX, there was only one single gene (*NDRG1*) in common for the 70 gene list in Mammaprint with our gene list separating pre- and postoperative samples. *NDRG1* (N-myc downstream-regulated gene 1) is a member of the N-myc downregulated gene family which belongs to the alpha/beta hydrolase superfamily. The protein encoded by this gene is a cytoplasmic protein involved in stress responses, hormone responses, cell growth, and differentiation. The encoded protein is necessary for p53-mediated caspase activation and apoptosis. Expression of this gene may be a prognostic indicator for several types of cancer (provided by RefSeq, May 2012). The gene is significantly upregulated in the postoperative samples of the present study. It is known to be induced by stress, through hypoxia [48], like many of the other genes mentioned above.

Low expression of *NDRG1* is correlated with poor clinical outcome in breast cancer [49]. It has also been shown that expression of *NDRG1* is downregulated upon estradiol stimulation, and its expression is correlated with favorable prognosis in breast cancer patients [50]. On the other hand, induction of its differentiation is considered a promising alternative or complementary to standard anticancer chemotherapy. One may speculate why this gene is upregulated in the postoperative samples. Stress is probably the cause, but since the gene is a positive predictive factor, can we then say that we place the tumor into a different prognostic group simply because of the stress of the procedure. Fotovati et al. [51] concluded that *NDRG1* could be used as a biomarker for differentiation of breast cancer for both diagnostic and therapeutic purposes. Still it is very important to be aware of at what material the gene is measured upon.

Our study shows the expression profiles found in the analyses of breast cancer tissue must be evaluated with caution. Different profiles may simply be result of differences in the surgical trauma and timing of when samples are taken, and not necessarily associated with tumor biology. 

## Supplementary Material

Supplementary Figure 1: Hierarchal clustering with all probes present on the array of the pre- and postoperative samples together with corresponding adjacent normal tissue shows overall similar expression profiles for the majority of the pre- and postoperative tumor pairs.
Supplementary Figure 2: Hierarchical clustering of the samples using the differently expressed genes (FDR < 2.5%) separates the majority of the preoperative tumors from the corresponding postoperative tumors.
Supplementary Figure 3: Molecular network showing the interaction between all the 228 differently expressed genes. The gene identifiers and corresponding expression values were uploaded into in the Ingenuity Pathway Analysis. Networks were then algorithmically generated based on their connectivity in Ingenuity's Knowledge Base. Molecules are represented as nodes, and the biological relationship between two nodes is represented as an edge (line). The intensity of the node color indicates the degree of up- (red) or down- (green) regulation. Nodes are displayed using various shapes that represent the functional class of the gene product.
Supplementary Table 1: Significantly differently expressed genes between pre- and postoperative samples. All probes with FDR < 2.5% ranked according to SAM. Systematic names shown in brackets.

## Figures and Tables

**Table 1:** Histopathological characteristics of the patients included in the study.

Case	Age	Tumour type*	TNM	Tumour size (cm)	Preoperative				Postoperative		
					Tumor content (%)	ER^†^ (%)	PGR^†^	HER2^†^	Tumor content (%)	ER^†^ (%)	PGR^†^
2	54	IDC	T2gr3N2M0	4.3	30	+ (>50)	−	+	30	−	−
3	42	IDC	T2gr3N0M0	4.8	40	−	−	−	60	−	−
4	67	IDC	T2gr2N1M0	3.5	50	+ (>50)	+	−	55	+ (>50)	+
5	82	IDC	T1cgr3N0M0	1.8	30	+ (>50)	+	−	40	+ (>50)	+
6	52	IDC	T2gr3N3M0	2.0	5	−	−	+	5	−	−
7	68	IDC	T1cgr3N1M0	1.3	35	+ (>50)	−	+	40	+ (>50)	+
8	76	IDC	T1cgr2N0M0	1.4	40	−	−	−	35	−	−
9	70	IDC	T1bgr1N0M0	0.9	25	+ (>50)	+	−	25	+ (>50)	+
10	77	IDC	T2gr3N1M0	2.6	50	+ (>50)	−	−	15	+ (>50)	−
11	61	IDC	T2gr3N1M0	2.5	50	−	−	+	40	−	−
13	79	IDC	T2gr3N0M0	2.3	45	+ (>10)	−	+	55	+ (>1)	−
14	70	IDC	T2gr2N0M0	2.3	35	+ (>50)	+	−	35	+ (>50)	+
15	68	ILC	T2gr2N0M0	2.5	50	+ (>50)	−		30	+ (>50)	+

*IDC: infiltrating ductal carcinoma; ILC: infiltrating lobular carcinoma.

^†^ER: oestrogen receptor status; PR: progesterone receptor status; HER2: HER2 receptor status.

**Table 2:** TaqMan assays used for validation qRT-PCR and correlation between the microarray and the qRT-PCR results. The *P* values given are for positive correlation. *r*: Pearson's product-moment correlation.

Gene	Array probe ID	TaqMan assay ID	Task	*r*	*P*
*18S *	NA	Hs99999901_s1	Endogenous control	NA	NA
*ACTB *	A_23_P31323 A_24_P226554 A_24_P226554 A_32_P137939	Hs99999903_m1	Endogenous control	0.904	1.7*E* − 4
*ANGEL2 *	A_24_P28622	Hs00404357_m1	Target	0.238	0.254
*CYP2D6 *	A_23_P143734 A_23_P155123	Hs02576167_m1	Target	0.262	0.232
*CYR61 *	A_23_P46426 A_24_P370946	Hs00155479_m1	Target	0.894	2.4*E* − 4
*DUSP1 *	A_23_P110712	Hs00610256_g1	Target	0.983	1.7*E* − 7
*DUSP9 *	A_24_P417189	Hs00154830_m1	Target	−0.630	0.965
*EVI2B *	A_23_P66694	Hs00272421_s1	Target	0.945	1.8*E* − 5
*FOSB *	A_23_P429998	Hs00171851_m1	Target	0.749	0.006
*GAPDH *	A_23_P13899	Hs99999905_m1	Endogenous control	0.632	0.025
*MALAT1 *	A_24_P497244	Hs00273907_s1	Target	0.332	0.174
*MAPK3 *	A_23_P37910	Hs00385075_m1	Target	−0.018	0.520
*NFR*κ*B*	A_23_P24485	Hs00196269_m1	Target	−0.169	0.680
*PTPRE *	A_24_P213494 A_24_P213503	Hs00369944_m1	Target	0.413	0.118
*RASD1 *	A_24_P348006 A_23_P118392	Hs02568415_s1	Target	0.797	0.003
*TMEM19 *	A_24_P358976	Hs00217586_m1	Target	0.248	0.245

**Table 3:** Selected genes that are differently expressed between pre- and postoperative samples.

Gene name	Agilent ID	Description	SAM			Fold Change	
			Called	FDR	*q*-val	Mean	Range
*CX3CL1 *	A_24_P390495	Chemokine (C-X3-C motif) ligand 1 (*CX3CL1*), mRNA (NM_002996)	21	0	0	2.99	0.90–4.09
*CYP2D6 *	A_23_P143734	Cytochrome P450, family 2, subfamily D, polypeptide 6 (*CYP2D6*), transcript variant 1, mRNA (NM_000106)	18	0	0	1.65	0.58–3.55
	A_23_P155123		103	2.08	1.55	1.51	0.46–3.38
*CYR61 *	A_23_P46426	Cysteine-rich, angiogenic inducer, 61 (*CYR61*), mRNA (NM_001554)	163	1.75	1.65	4.07	0.61–12.88
	A_24_P370946		196	1.82	1.79	5.51	0.36–22.80
*DUSP1 *	A_23_P110712	Dual specificity phosphatase 1 (*DUSP1*), mRNA (NM_004417)	217	2.3	2.20	3.18	0.49–12.69
*DUSP9 *	A_24_P417189	Dual specificity phosphatase 9 (*DUSP9*), mRNA (NM_001395)	7	0	0	2.12	1.15–2.88
*FOSB *	A_23_P429998	FBJ murine osteosarcoma viral oncogene homolog B (*FOSB*), transcript variant 1, mRNA (NM_006732)	203	2.11	2.01	2.79	0.96–24.26
*MALAT1 *	A_24_P497244	Metastasis associated lung adenocarcinoma transcript 1 (nonprotein coding) (*MALAT1*), noncoding RNA (NR_002819)	3	0	0	3.54	1.31–9.97
*MAPK3 *	A_23_P37910	Mitogen-activated protein kinase 3 (*MAPK3*), transcript variant 1, mRNA (NM_002746)	42	1.70	1.30	2.01	0.40–2.55
*NFR*κ*B*	A_23_P24485	nuclear factor related to kappaB binding protein (*NFR*κ*B*), transcript variant 2, mRNA (NM_006165)	183	1.95	1.79	1.61	0.63–3.25
*RAB17 *	A_23_P5778	*RAB17*, member RAS oncogene family (*RAB17*), mRNA (NM_022449)	10	0	0	1.84	0.23–4.36
*RASAL1 *	A_23_P139600	RAS protein activator like 1 (GAP1 like) (*RASAL1*), mRNA (NM_004658)	140	2.04	1.65	1.46	0.38–2.41
*RASD1 *	A_23_P118392	RAS, dexamethasone-induced 1 (*RASD1*), mRNA (NM_016084)	105	2.04	1.55	3.14	0.88–21.55
	A_24_P348006		27	0	0	2.69	0.85–12.90
*RHOB *	A_23_P51136	ras homolog gene family, member B (*RHOB*), mRNA (NM_004040)	16	0	0	1.99	0.51–2.96
*RHOU *	A_23_P114814	ras homolog gene family, member U (*RHOU*), mRNA (NM_021205)	122	1.75	1.55	2.68	0.71–3.65
*RHOV *	A_23_P117912	Rho-related GTP-binding protein RhoV (Wnt-1 responsive Cdc42 homolog 2)(WRCH-2)(CDC42-like GTPase 2)(GTP-binding protein-like 2) (Rho GTPase-like protein ARHV) (ENST00000220507)	184	1.94	1.79	1.60	0.25–5.45

**Table 4:** Gene functional classification (DAVID) of the differently expressed genes.

Gene name	Agilent ID	Description
Gene group 1		Enrichment score: 2.11
Kinase/phosphatase		

*DCAKD *	A_24_P58331	Dephospho-CoA kinase domain containing
*SIK2 *	A_23_P138957	Salt-inducible kinase 2
*ITPKC *	A_23_P208369	Inositol 1,4,5-trisphosphate 3-kinase C
*DAK *	A_23_P36129	Dihydroxyacetone kinase 2 homolog (*S. cerevisiae*)
*RIPK4 *	A_23_P211267	Receptor-interacting serine-threonine kinase 4
*CHKA *	A_23_P136135	Choline kinase alpha
*DDR1 *	A_23_P93311, A_24_P367289	Discoidin domain receptor tyrosine kinase 1
*STK35 *	A_24_P940537	Serine/threonine kinase 35
*ACTR3B *	A_23_P123193	ARP3 actin-related protein 3 homolog B (yeast)
*INO80 *	A_24_P39454	INO80 homolog (*S. cerevisiae*)
*EPHA1 *	A_23_P157333	EPH receptor A1
*BCR *	A_24_P15270	Breakpoint cluster region
*CAMK1D *	A_23_P124252	Calcium/calmodulin-dependent protein kinase ID
*HISPPD2A *	A_23_P205818	Histidine acid phosphatase domain containing 2A

Gene group 2		Enrichment score: 1.35
Ras		

*RHOB *	A_23_P51136	Ras homolog gene family, member B
*RHOV *	A_23_P117912	Ras homolog gene family, member V
*RASD1 *	A_24_P348006, A_23_P118392	RAS, dexamethasone-induced 1
*RAB17 *	A_23_P5778	RAB17, member RAS oncogene family
*RHOU *	A_23_P114814	Ras homolog gene family, member U

Gene group 3		Enrichment score: 1.26
Negative regulation of transcription		

*ARID5B *	A_23_P97871	AT rich interactive domain 5B (MRF1-like)
*COBRA1 *	A_23_P148150	Cofactor of BRCA1
*TH1L *	A_24_P222126	TH1-like (Drosophila)
*FOXD3 *	A_23_P46560	Forkhead box D3
*EID2 *	A_23_P365844	EP300 interacting inhibitor of differentiation 2

Gene group 4		Enrichment score: 0.42
Transmembrane		

*PQLC1 *	A_24_P181677	PQ loop repeat containing 1
*RNF215 *	A_32_P420563	Ring finger protein 215
*KIAA1305 *	A_23_P129005	KIAA1305
*TMEM49 *	A_32_P9753	Transmembrane protein 49
*F11R *	A_24_P319369	F11 receptor
*RBM8A *	A_23_P305335	Gonadotropin-releasing hormone (type 2) receptor 2
*KIAA0922 *	A_23_P257250	KIAA0922
*TSPAN12 *	A_23_P145984	Tetraspanin 12
*DGCR2 *	A_24_P125881	DiGeorge syndrome critical region gene 2
*PCDH1 *	A_23_P213359	Protocadherin 1
*LMBRD2 *	A_32_P8952	LMBR1 domain containing 2
*GPR65 *	A_23_P14564	G protein-coupled receptor 65
*EVI2B *	A_23_P66694	Ecotropic viral integration site 2B
*RTF1 *	A_24_P93741	RFT1 homolog (*S. cerevisiae*)
*TMEM19 *	A_24_P358976	Transmembrane protein 19
*GPR155 *	A_23_P335958	G protein-coupled receptor 155
*OSMR *	A_24_P145134	Oncostatin M receptor
*TMEM97 *	A_32_P201521	Transmembrane protein 97
*PTPRE *	A_24_P213503, A_24_P213494	Protein tyrosine phosphatase, receptor type, E

**Table 5:** Intrinsic subtypes of the tumours. Samples with all correlations < 0.1 were not assigned to any subtype.

Patient no.	Sample type	Score						Subtype
		LumA	LumB	ERBB2	Normal	Basal	Max
2	Preoperative	−0.050	0.231	0.103	−0.191	−0.034	0.231	Luminal B
	Postoperative	−0.081	0.193	0.010	0.011	0.062	0.193	Luminal B
3	Preoperative	−0.465	0.213	0.215	−0.061	0.616	0.616	Basal-like
	Postoperative	−0.454	0.177	0.190	0.010	0.668	0.668	Basal-like
4	Preoperative	0.064	0.152	−0.058	−0.268	−0.085	0.152	Luminal B
	Postoperative	0.085	0.063	0.017	−0.271	−0.227	0.085	NA
5	Preoperative	−0.001	0.032	−0.028	0.014	−0.116	0.032	NA
	Postoperative	−0.050	0.088	−0.061	−0.056	−0.079	0.088	NA
6	Preoperative	−0.454	0.067	0.477	−0.017	0.374	0.477	ERBB2
	Postoperative	−0.576	0.096	0.531	−0.015	0.410	0.531	ERBB2
7	Preoperative	0.234	0.031	−0.165	−0.212	−0.224	0.234	Luminal A
	Postoperative	0.123	−0.115	−0.106	0.067	−0.185	0.123	Luminal A
8	Preoperative	−0.117	−0.011	0.222	−0.066	0.031	0.222	ERBB2
	Postoperative	−0.066	−0.060	0.144	−0.065	−0.039	0.144	ERBB2
9	Preoperative	−0.127	−0.189	0.149	0.188	0.055	0.188	Normal-like
	Postoperative	−0.040	−0.220	0.077	0.266	0.005	0.266	Normal-like
10	Preoperative	0.032	0.039	−0.025	−0.085	−0.138	0.039	NA
	Postoperative	−0.003	0.218	−0.082	−0.201	−0.035	0.218	Luminal B
11	Preoperative	−0.269	0.260	0.242	−0.163	0.217	0.260	Luminal B
	Postoperative	−0.147	0.292	0.132	−0.174	0.086	0.292	Luminal B
13	Preoperative	−0.266	**0.130**	0.166	−0.002	**0.211**	0.211	Basal-like*
	Postoperative	−0.105	**0.246**	0.089	−0.197	**0.007**	0.246	Luminal B*
14	Preoperative	**0.169**	−0.111	−0.142	**−0.017**	−0.170	0.169	Luminal A*
	Postoperative	**0.068**	−0.175	−0.092	**0.196**	−0.026	0.196	Normal-like*
15	Preoperative	0.356	−0.013	−0.328	−0.188	−0.338	0.356	Luminal A
	Postoperative	0.223	−0.065	−0.131	−0.094	−0.349	0.223	Luminal A

*Different subtypes in the pre- and postoperative samples.

## References

[1] Townsend CM, Beauchamp RD, Evers BM (2004). *Sabiston Textbook of Surgery: The Biological Basis of Modern Surgical Practice*.

[2] Veronesi U, Paganelli G, Viale G (2003). A randomized comparison of sentinel-node biopsy with routine axillary dissection in breast cancer. *The New England Journal of Medicine*.

[3] Perou CM, Sørile T, Eisen MB (2000). Molecular portraits of human breast tumours. *Nature*.

[4] Sørlie T, Tibshirani R, Parker J (2003). Repeated observation of breast tumor subtypes in independent gene expression data sets. *Proceedings of the National Academy of Sciences of the United States of America*.

[5] Sotiriou C, Neo SY, McShane LM (2003). Breast cancer classification and prognosis based on gene expression profiles from a population-based study. *Proceedings of the National Academy of Sciences of the United States of America*.

[6] Van’t Veer LJ, Dai H, van de Vijver MJ (2002). Gene expression profiling predicts clinical outcome of breast cancer. *Nature*.

[7] Zhao H, Langerød A, Ji Y (2004). Different gene expression patterns in invasive lobular and ductal carcinomas of the breast. *Molecular Biology of the Cell*.

[8] van de Vijver MJ, He YD, Van ’T Veer LJ (2002). A gene-expression signature as a predictor of survival in breast cancer. *The New England Journal of Medicine*.

[9] Wang Y, Klijn JGM, Zhang Y (2005). Gene-expression profiles to predict distant metastasis of lymph-node-negative primary breast cancer. *The Lancet*.

[10] Weigelt B, Peterse JL, Van’t Veer LJ (2005). Breast cancer metastasis: markers and models. *Nature Reviews Cancer*.

[11] Wong V, Wang DY, Warren K (2008). The effects of timing of fine needle aspiration biopsies on gene expression profiles in breast cancers. *BMC Cancer*.

[12] de Cecco L, Musella V, Veneroni S (2009). Impact of biospecimens handling on biomarker research in breast cancer. *BMC Cancer*.

[13] Trifirò G, Viale G, Gentilini O, Lavinia Travaini L, Paganelli G (2004). Sentinel node detection in pre-operative axillary staging. *European Journal of Nuclear Medicine and Molecular Imaging*.

[14] Wei JS, Khan J, Bowtell D, Sambrook J (2002). Purification of total RNA from mammalian cells and tissues. *DNA Microarrays: A Molecular Cloning Manual*.

[15] Dysvik B, Jonassen I (2001). J-Express: exploring gene expression data using Java. *Bioinformatics*.

[16] Huang DW, Sherman BT, Lempicki RA (2009). Bioinformatics enrichment tools: paths toward the comprehensive functional analysis of large gene lists. *Nucleic Acids Research*.

[17] Huang DW, Sherman BT, Lempicki RA (2009). Systematic and integrative analysis of large gene lists using DAVID bioinformatics resources. *Nature Protocols*.

[18] Livak KJ, Schmittgen TD (2001). Analysis of relative gene expression data using real-time quantitative PCR and the 2-ΔΔCT method. *Methods*.

[19] Tusher VG, Tibshirani R, Chu G (2001). Significance analysis of microarrays applied to the ionizing radiation response. *Proceedings of the National Academy of Sciences of the United States of America*.

[20] Haibe-Kains B, Desmedt C, Piette F (2008). Comparison of prognostic gene expression signatures for breast cancer. *BMC Genomics*.

[21] Sotiriou C, Pusztai L (2009). Gene-expression signatures in breast cancer. *The New England Journal of Medicine*.

[22] Weigelt B, Baehner FL, Reis-Filho JS (2010). The contribution of gene expression profiling to breast cancer classification, prognostication and prediction: a retrospective of the last decade. *Journal of Pathology*.

[23] Cardoso F, Piccart-Gebhart M, Van’t Veer L, Rutgers E (2007). The MINDACT trial: the first prospective clinical validation of a genomic tool. *Molecular Oncology*.

[24] Paik S (2007). Development and clinical utility of a 21-gene recurrence score prognostic assay in patients with early breast cancer treated with tamoxifen. *Oncologist*.

[25] Ma SF, Grigoryev DN, Taylor AD (2005). Bioinformatic identification of novel early stress response genes in rodent models of lung injury. *American Journal of Physiology*.

[26] Ruel M, Bianchi C, Khan TA (2003). Gene expression profile after cardiopulmonary bypass and cardioplegic arrest. *Journal of Thoracic and Cardiovascular Surgery*.

[27] Tarozzo G, Campanella M, Ghiani M, Bulfone A, Beltramo M (2002). Expression of fractalkine and its receptor, CX3CR1, in response to ischaemia-reperfusion brain injury in the rat. *European Journal of Neuroscience*.

[28] Morita K, Saito T, Ohta M (2005). Expression analysis of psychological stress-associated genes in peripheral blood leukocytes. *Neuroscience Letters*.

[29] Bièche I, Lerebours F, Tozlu S, Espie M, Marty M, Lidereau R (2004). Molecular profiling of inflammatory breast cancer: identification of a poor-prognosis gene expression signature. *Clinical Cancer Research*.

[30] Langer S, Singer CF, Hudelist G (2006). Jun and Fos family protein expression in human breast cancer: correlation of protein expression and clinicopathological parameters. *European Journal of Gynaecological Oncology*.

[31] Lu C, Shen Q, DuPré E, Kim H, Hilsenbeck S, Brown PH (2005). cFos is critical for MCF-7 breast cancer cell growth. *Oncogene*.

[32] Chen CC, Lau LF (2009). Functions and mechanisms of action of CCN matricellular proteins. *International Journal of Biochemistry and Cell Biology*.

[33] Babic AM, Kireeva ML, Kolesnikova TV, Lau LF (1998). CYR61, a product of a growth factor-inducible immediate early gene, promotes angiogenesis and tumor growth. *Proceedings of the National Academy of Sciences of the United States of America*.

[34] Sampath D, Winneker RC, Zhang Z (2001). Cyr61, a member of the CCN family, is required for MCF-7 cell proliferation: regulation by 17*β*-estradiol and overexpression in human breast cancer. *Endocrinology*.

[35] Folgueira MAAK, Brentani H, Carraro DM (2009). Gene expression profile of residual breast cancer after doxorubicin and cyclophosphamide neoadjuvant chemotherapy. *Oncology Reports*.

[36] Wang HY, Cheng Z, Malbon CC (2003). Overexpression of mitogen-activated protein kinase phosphatases MKP1, MKP2 in human breast cancer. *Cancer Letters*.

[37] Liu YX, Wang J, Guo J, Wu J, Lieberman HB, Yin Y (2008). DUSP1 is controlled by p53 during the cellular response to oxidative stress. *Molecular Cancer Research*.

[38] Symmans WF, Ayers M, Clark EA (2003). Total RNA yield and microarray gene expression profiles from fine-needle aspiration biopsy and core-needle biopsy samples of breast carcinoma. *Cancer*.

[39] Benetkiewicz M, Piotrowski A, de Ståhl TD (2006). Chromosome 22 array-CGH profiling of breast cancer delimited minimal common regions of genomic imbalances and revealed frequent intra-tumoral genetic heterogeneity. *International Journal of Oncology*.

[40] Geyer FC, Weigelt B, Natrajan R (2010). Molecular analysis reveals a genetic basis for the phenotypic diversity of metaplastic breast carcinomas. *Journal of Pathology*.

[41] Sørlie T, Perou CM, Tibshirani R (2001). Gene expression patterns of breast carcinomas distinguish tumor subclasses with clinical implications. *Proceedings of the National Academy of Sciences of the United States of America*.

[42] Paik S, Shak S, Tang G (2004). A multigene assay to predict recurrence of tamoxifen-treated, node-negative breast cancer. *The New England Journal of Medicine*.

[43] Giricz O, Calvo V, Pero SC (2012). GRB7 is required for triple-negative breast cancer cell invasion and survival. *Breast Cancer Research and Treatment*.

[44] Sparano JA, Goldstein LJ, Childs BH (2011). Relationship between quantitative GRB7 RNA expression and recurrence after adjuvant anthracycline chemotherapy in triple-negative breast cancer. *Clinical Cancer Research*.

[45] Margolis B, Silvennoinen O, Comoglio F (1992). High-efficiency expression/cloning of epidermal growth factor-receptor- binding proteins with Src homology 2 domains. *Proceedings of the National Academy of Sciences of the United States of America*.

[46] Stein D, Wu J, Fuqua SAW (1994). The SH2 domain protein GRB-7 is co-amplified, overexpressed and in a tight complex with HER2 in breast cancer. *EMBO Journal*.

[47] Yokote K, Margolis B, Heldin CH, Claesson-Welsh L (1996). Grb7 is a downstream signaling component of platelet-derived growth factor and *β*-receptors. *The Journal of Biological Chemistry*.

[48] Cangul H (2004). Hypoxia upregulates the expression of the NDRG1 gene leading to its overexpression in various human cancers. *BMC Genetics*.

[49] Bandyopadhyay S, Pai SK, Hirota S (2004). Role of the putative tumor metastasis suppressor gene Drg-1 in breast cancer progression. *Oncogene*.

[50] Fotovati A, Fujii T, Yamaguchi M (2006). 17*β*-estradiol induces down-regulation of Cap43/NDRG1/Drg-1, a putative differentiation-related and metastasis suppressor gene, in human breast cancer cells. *Clinical Cancer Research*.

[51] Fotovati A, Abu-Ali S, Kage M, Shirouzu K, Yamana H, Kuwano M (2011). N-myc downstream-regulated gene 1 (NDRG1) a differentiation marker of human breast cancer. *Pathology and Oncology Research*.

