# Supplementary material for: Molecular Profiles of Pre- and Postoperative Breast Cancer Tumours Reveal Differentially Expressed Genes
Source: ISRN Oncol. 2012 Nov 26;2012:450267. doi: 10.5402/2012/450267 (PMC3513735; doi:10.5402/2012/450267)
Supplement: Supplementary file 1 — Supplementary Figure 1: Hierarchal clustering with all probes present on the array of the pre- and postoperative samples together with corresponding adjacent normal tissue shows overall similar expression profiles for the majority of the pre- and postoperative tumor pairs. Supplementary Figure 2: Hierarchical clustering of the samples using the differently expressed genes (FDR < 2.5%) separates the majority of the preoperative tumors from the corresponding postoperative tumors. Supplementary Figure 3: Molecular network showing the interaction between all the 228 differently expressed genes. The gene identifiers and corresponding expression values were uploaded into in the Ingenuity Pathway Analysis. Networks were then algorithmically generated based on their connectivity in Ingenuity's Knowledge Base. Molecules are represented as nodes, and the biological relationship between two nodes is represented as an edge (line). The intensity of the node color indicates the degree of up- (red) or down- (green) regulation. Nodes are displayed using various shapes that represent the functional class of the gene product. Supplementary Table 1: Significantly differently expressed genes between pre- and postoperative samples. All probes with FDR < 2.5% ranked according to SAM. Systematic names shown in brackets. [file 450267.f1.pdf]

Supplementary table 1 Significantly differently expressed genes between pre- and postoperative samples. All probes with FDR < 2.5% ranked according to SAM. Systematic names shown in brackets.

Gene Name	Agilent ID	Description	SAM				Fold change	
			Called	FSN	FDR	q-val	mean	Range
PACSIN2	A_32_P38145	cDNA DKFZp434H1130 (from clone DKFZp434H1130), [AL136845]	1	0	0	0	2,03	0,53-2,55
PLA2G3	A_23_P17814	phospholipase A2, group III (PLA2G3), mRNA [NM_015715]	2	0	0	0	2,2	0,11-4,07
MALAT1	A_24_P497244	metastasis associated lung adenocarcinoma transcript 1 (non-protein coding) (MALAT1), non-coding RNA [NR_002819]	3	0	0	0	3,54	1,31-9,97
DAK	A_23_P36129	dihydroxyacetone kinase 2 homolog (S, cerevisiae) (DAK), mRNA [NM_015533]	4	0	0	0	1,71	0,36-2,65
HISPPD2A	A_23_P205818	histidine acid phosphatase domain containing 2A (HISPPD2A), transcript variant 4, mRNA [NM_001024463]	5	0	0	0	1,71	0,76-3,01
PCDH1	A_23_P213359	protocadherin 1 (PCDH1), transcript variant 1, mRNA [NM_002587]	6	0	0	0	1,64	0,38-3,45
DUSP9	A_24_P417189	dual specificity phosphatase 9 (DUSP9), mRNA [NM_001395]	7	0	0	0	2,12	1,15-2,88
DFNB31	A_24_P376129	cDNA FLJ31628 fis, clone NT2RI2003344, [AK056190]	8	0	0	0	3,15	1,30-5,69
KIAA1305	A_23_P129005	KIAA1305 (KIAA1305), mRNA [NM_025081]	9	0	0	0	1,65	1,11-2,19
RAB17	A_23_P5778	RAB17, member RAS oncogene family (RAB17), mRNA [NM_022449]	10	0	0	0	1,84	0,23-4,36
NCK2	A_23_P50962	NCK adaptor protein 2 (NCK2), transcript variant 1, mRNA [NM_003581]	11	0	0	0	1,9	1,00-4,13
LMBRD2	A_32_P8952	LMBR1 domain containing 2 (LMBRD2), mRNA [NM_001007527]	12	0	0	0	1,79	1,08-3,63
THC2610143	A_32_P124580	aa43f10,r1 Soares_NhHMPu_S1 Homo sapiens cDNA clone IMAGE:823723 5', mRNA sequence [AA490192]	13	0	0	0	1,61	1,01-2,43
HES1	A_23_P6596	hairy and enhancer of split 1, (Drosophila) (HES1), mRNA [NM_005524]	14	0	0	0	1,87	0,77-4,18
STARD13	A_24_P210420	StAR-related lipid transfer (START) domain containing 13 (STARD13), transcript variant alpha, mRNA [NM_178006]	15	0	0	0	1,44	0,28-2,15
RHOB	A_23_P51136	ras homolog gene family, member B (RHOB), mRNA [NM_004040]	16	0	0	0	1,99	0,51-2,96
AK075052	A_24_P726399	cDNA FLJ90571 fis, clone OVARC1001725, highly similar to Homo sapiens patched related protein TRC8 (TRC8) gene, [AK075052]	17	0	0	0	2,52	0,88-2,97
CYP2D6	A_23_P143734	cytochrome P450, family 2, subfamily D, polypeptide 6 (CYP2D6), transcript variant 1, mRNA [NM_000106]	18	0	0	0	1,65	0,58-3,55
C10orf10	A_24_P329795	chromosome 10 open reading frame 10 (C10orf10), mRNA [NM_007021]	19	0	0	0	2,08	0,88-18,37
EEA1	A_23_P76159	early endosome antigen 1 (EEA1), mRNA [NM_003566]	20	0	0	0	1,4	0,33-1,84
CX3CL1	A_24_P390495	chemokine (C-X3-C motif) ligand 1 (CX3CL1), mRNA [NM_002996]	21	0	0	0	2,99	0,90-4,09

Gene Name	Agilent ID	Description	SAM				Fold change	
			Called	FSN	FDR	q-val	mean	Range
BC039457	A_24_P368023	cDNA clone IMAGE:5312122, [BC039457]	22	0	0	0	1,56	0,95-2,09
A_23_P206135	A_23_P206135	Unknown	23	0	0	0	1,71	0,51-2,51
ANAPC2	A_23_P253068	anaphase promoting complex subunit 2 (ANAPC2), mRNA [NM_013366]	24	0	0	0	1,74	0,45-3,58
BCR	A_24_P15270	breakpoint cluster region (BCR), transcript variant 1, mRNA [NM_004327]	25	0	0	0	1,6	0,63-2,90
A_24_P593733	A_24_P593733	Unknown	26	0	0	0	1,9	0,55-3,66
RASD1	A_24_P348006	RAS, dexamethasone-induced 1 (RASD1), mRNA [NM_016084]	27	0	0	0	2,69	0,85-12,90
PQLC1	A_24_P181677	PQ loop repeat containing 1 (PQLC1), mRNA [NM_025078]	28	0	0	0	1,6	0,97-2,25
CIITA	A_23_P353478	CIITA-8 MHC class II transactivator CIITA mRNA, complete cds, [U18259]	29	0,713	2,458	1,485	0,52	0,15-3,01
ANKRD35	A_23_P325690	ankyrin repeat domain 35 (ANKRD35), mRNA [NM_144698]	30	0,713	2,377	1,296	1,93	0,11-3,82
LRRC1	A_23_P377350	cDNA FLJ11834 fis, clone HEMBA1006583, weakly similar to Drosophila melanogaster Scribble (scrib) mRNA, [AK021896]	31	0,713	2,3	1,296	1,79	0,99-4,69
BX110908	A_32_P4172	Soares_testis_NHT Homo sapiens cDNA clone IMAGp998G162577, mRNA sequence [BX110908]	32	0,713	2,228	1,485	0,54	0,19-4,04
C21orf58	A_23_P132139	chromosome 21 open reading frame 58 (C21orf58), mRNA [NM_058180]	33	0,713	2,16	1,296	1,96	0,68-3,73
AK098372	A_24_P238649	cDNA FLJ25506 fis, clone CBR05185, [AK098372]	34	0,713	2,097	1,296	1,66	0,42-5,33
AB040974	A_24_P940218	mRNA for KIAA1541 protein, partial cds, [AB040974]	35	0,713	2,037	1,296	1,91	1,20-6,61
FBXW4P1	A_23_P252603	F-box protein Fbw3 (FBW3) mRNA, complete cds, [AF174606]	36	0,713	1,98	1,296	2,1	0,39-5,90
NCAPG2	A_23_P168747	non-SMC condensin II complex, subunit G2 (NCAPG2), mRNA [NM_017760]	37	0,713	1,927	1,296	2,81	0,77-8,76
ADAMTS1	A_23_P211039	ADAM metallopeptidase with thrombospondin type 1 motif, 1 (ADAMTS1), mRNA [NM_006988]	38	0,713	1,876	1,296	2,45	0,80-11,37
THC2505349	A_32_P48856	ALU6_HUMAN (P39193) Alu subfamily SP sequence contamination warning entry, partial (13%) [THC2505349]	39	0,713	1,828	1,485	0,77	0,70-1,67
TRIB1	A_23_P123503	tribbles homolog 1 (Drosophila) (TRIB1), mRNA [NM_025195]	40	0,713	1,782	1,296	1,69	0,82-3,72
LOC392335	A_32_P223173	PREDICTED: Homo sapiens misc_RNA (LOC392335), miscRNA [XR_037043]	41	0,713	1,739	1,296	1,72	0,52-3,00
MAPK3	A_23_P37910	mitogen-activated protein kinase 3 (MAPK3), transcript variant 1, mRNA [NM_002746]	42	0,713	1,698	1,296	2,01	0,40-2,55
C4orf23	A_24_P320171	chromosome 4 open reading frame 23 (C4orf23), transcript variant 2, mRNA [NM_152544]	43	0,713	1,658	1,296	1,87	0,50-4,59
TMEM49	A_32_P9753	transmembrane protein 49, mRNA (cDNA clone IMAGE:4295896), with apparent retained intron, [BC024020]	44	0,713	1,62	1,296	2,09	1,10-5,02
PTPRE	A_24_P213494	protein tyrosine phosphatase, receptor type, E (PTPRE), transcript variant 1, mRNA [NM_006504]	45	0,713	1,584	1,485	0,38	0,16-1,10
SH2D3A	A_24_P262728	SH2 domain containing 3A (SH2D3A), mRNA [NM_005490]	46	0,713	1,55	1,296	1,5	0,30-2,74
BC036645	A_24_P570806	cDNA clone IMAGE:4814437, [BC036645]	47	0,713	1,517	1,296	1,57	0,74-2,54

Gene Name	Agilent ID	Description	SAM				Fold change	
			Called	FSN	FDR	q-val	mean	Range
AA455656	A_32_P92415	aa22e03,s1 NCI_CGAP_GCB1 Homo sapiens cDNA clone IMAGE:814012 3', mRNA sequence [AA455656]	48	0,713	1,485	1,485	0,6	0,43-1,81
RXRB	A_23_P59179	retinoid X receptor, beta (RXRB), mRNA [NM_021976]	49	0,713	1,455	1,296	1,97	0,70-3,71
ECHDC2	A_24_P225477	enoyl Coenzyme A hydratase domain containing 2 (ECHDC2), mRNA [NM_018281]	50	0,713	1,426	1,296	1,85	0,59-3,37
EDC4	A_24_P399009	enhancer of mRNA decapping 4 (EDC4), mRNA [NM_014329]	51	0,713	1,398	1,296	1,77	0,59-2,60
CASKIN2	A_24_P48187	CASK interacting protein 2 (CASKIN2), transcript variant 1, mRNA [NM_020753]	52	0,713	1,371	1,296	1,77	0,82-2,78
HSPG2	A_23_P23191	heparan sulfate proteoglycan 2 (HSPG2), mRNA [NM_005529]	53	0,713	1,345	1,296	2,01	0,45-2,77
SF3B3	A_23_P135914	splicing factor 3b, subunit 3, 130kDa (SF3B3), mRNA [NM_012426]	54	0,713	1,32	1,296	1,4	0,92-2,85
CTA-216E10,6	A_23_P132341	hypothetical FLJ23584 (FLJ23584), mRNA [NM_001142964]	55	0,713	1,296	1,296	1,37	0,45-3,12
PRDM11	A_23_P13057	PR domain containing 11 (PRDM11), mRNA [NM_020229]	56	1,426	2,546	1,44	1,65	0,50-2,84
A_24_P900555	A_24_P900555	Unknown	57	1,426	2,502	1,44	1,59	0,72-3,87
ITPKC	A_23_P208369	inositol 1,4,5-trisphosphate 3-kinase C (ITPKC), mRNA [NM_025194]	58	1,426	2,458	1,44	1,47	0,71-2,04
ZZEF1	A_23_P130107	cDNA FLJ10821 fis, clone NT2RP4001057, [AK001683]	59	1,426	2,417	1,44	1,48	1,04-2,55
AZI1	A_24_P66522	5-azacytidine induced 1 (AZI1), transcript variant 1, mRNA [NM_014984]	60	1,426	2,377	1,44	1,76	0,28-3,90
C1orf183	A_24_P336577	chromosome 1 open reading frame 183 (C1orf183), transcript variant 1, mRNA [NM_019099]	61	1,426	2,338	1,44	1,65	1,02-2,14
AK123264	A_24_P930088	cDNA FLJ41270 fis, clone BRAMY2036387, [AK123264]	62	1,426	2,3	1,55	0,27	0,07-1,41
SIK2	A_23_P138957	salt-inducible kinase 2 (SIK2), mRNA [NM_015191]	63	1,426	2,263	1,44	2,3	0,99-10,33
FLJ38359	A_24_P163009	cDNA FLJ38359 fis, clone FEBRA2000321, [AK095678]	64	1,426	2,228	1,44	3,04	0,80-25,23
HEATR5B	A_32_P389118	HEAT repeat containing 5B (HEATR5B), mRNA [NM_019024]	65	1,426	2,194	1,44	1,33	1,05-2,25
SC4MOL	A_23_P110184	sterol-C4-methyl oxidase-like (SC4MOL), transcript variant 1, mRNA [NM_006745]	66	1,426	2,16	1,44	1,76	0,52-2,25
ERO1LB	A_23_P347618	ERO1-like beta (S, cerevisiae) (ERO1LB), mRNA [NM_019891]	67	1,426	2,128	1,44	1,54	0,57-2,29
TAX1BP3	A_23_P38468	Tax1 (human T-cell leukemia virus type I) binding protein 3 (TAX1BP3), mRNA [NM_014604]	68	1,426	2,097	1,44	1,39	0,45-2,49
MBD6	A_32_P411592	methyl-CpG binding domain protein 6 (MBD6), mRNA [NM_052897]	69	1,426	2,067	1,44	1,76	0,72-3,95
WDR26	A_24_P42803	WD repeat domain 26 (WDR26), transcript variant 1, mRNA [NM_025160]	70	1,426	2,037	1,44	1,97	0,62-2,74
A_32_P82119	A_32_P82119	Unknown	71	1,426	2,008	1,44	1,73	0,82-4,26
MYO10	A_24_P46357	myosin X (MYO10), mRNA [NM_012334]	72	1,426	1,98	1,44	2,68	1,08-5,29
GOLGA2	A_24_P910297	golgi autoantigen, golgin subfamily a, 2 (GOLGA2), mRNA [NM_004486]	73	1,426	1,953	1,44	1,75	0,73-2,63

Gene Name	Agilent ID	Description	SAM				Fold change	
			Called	FSN	FDR	q-val	mean	Range
MTMR14	A_24_P388502	myotubularin related protein 14 (MTMR14), transcript variant 3, mRNA [NM_022485]	74	1,426	1,927	1,44	1,37	0,88-4,14
TMEM97	A_32_P201521	transmembrane protein 97 (TMEM97), mRNA [NM_014573]	75	1,426	1,901	1,55	0,58	0,27-1,03
FLJ36031	A_23_P377982	hypothetical protein FLJ36031 (FLJ36031), mRNA [NM_175884]	76	1,426	1,876	1,44	2,06	0,63-5,49
FOXD3	A_23_P46560	forkhead box D3 (FOXD3), mRNA [NM_012183]	77	1,426	1,852	1,44	1,61	0,84-2,49
GPR155	A_23_P335958	G protein-coupled receptor 155 (GPR155), transcript variant 9, mRNA [NM_001033045]	78	1,426	1,828	1,55	0,65	0,31-1,62
THC2537951	A_32_P217330	ALU1_HUMAN (P39188) Alu subfamily J sequence contamination warning entry, partial (27%) [THC2537674]	79	1,426	1,805	1,44	1,52	0,95-2,22
DIS3L2	A_23_P113888	DIS3 mitotic control homolog (S, cerevisiae)-like 2 (DIS3L2), mRNA [NM_152383]	80	1,426	1,782	1,44	1,49	0,74-2,27
THC2708124	A_32_P73304	Unknown	81	1,426	1,76	1,55	0,65	0,26-2,75
AK024470	A_24_P84370	mRNA for FLJ00063 protein, partial cds, [AK024470]	82	1,426	1,739	1,44	1,92	1,12-6,35
WASF2	A_32_P41461	WAS protein family, member 2 (WASF2), mRNA [NM_006990]	83	1,426	1,718	1,55	0,59	0,25-3,08
INPPL1	A_23_P36322	inositol polyphosphate phosphatase-like 1 (INPPL1), mRNA [NM_001567]	84	1,426	1,698	1,44	1,61	0,71-2,16
LOC344382	A_24_P135579	PREDICTED: Homo sapiens similar to unr-interacting protein (LOC344382), mRNA [XM_293026]	85	1,426	1,678	1,44	1,24	1,05-2,29
BC031320	A_24_P523061	cDNA clone IMAGE:5278682, [BC031320]	86	1,426	1,658	1,44	1,32	0,78-1,98
ZW10	A_23_P64204	ZW10, kinetochore associated, homolog (Drosophila) (ZW10), mRNA [NM_004724]	87	1,426	1,639	1,44	1,35	0,83-2,69
COBRA1	A_23_P148150	cofactor of BRCA1 (COBRA1), mRNA [NM_015456]	88	1,426	1,62	1,44	1,33	0,65-1,86
SUZ12P	A_24_P889103	full-length cDNA clone CS0DC012YL18 of Neuroblastoma Cot 25-normalized of Homo sapiens (human), [CR597846]	89	1,426	1,602	1,44	1,73	0,91-2,96
GRB7	A_23_P163992	growth factor receptor-bound protein 7 (GRB7), transcript variant 1, mRNA [NM_005310]	90	1,426	1,584	1,44	1,62	0,55-3,23
DTNB	A_24_P216421	dystrobrevin, beta (DTNB), transcript variant 3, mRNA [NM_033148]	91	1,426	1,567	1,44	1,66	0,23-2,53
A_32_P194182	A_32_P194182	Unknown	92	1,426	1,55	1,55	0,68	0,43-1,52
F11R	A_24_P319369	F11 receptor (F11R), mRNA [NM_016946]	93	1,426	1,533	1,44	1,48	0,21-2,39
BC016384	A_24_P677890	clone IMAGE:4703872, mRNA, [BC016384]	94	1,426	1,517	1,44	1,35	1,07-3,74
ALS2CL	A_32_P420009	ALS2 C-terminal like (ALS2CL), transcript variant 1, mRNA [NM_147129]	95	1,426	1,501	1,44	1,82	0,60-2,76
SLC25A4	A_24_P206047	solute carrier family 25 (mitochondrial carrier; adenine nucleotide translocator), member 4 (SLC25A4), nuclear gene encoding mitochondrial protein, mRNA [NM_001151]	96	1,426	1,485	1,44	1,48	0,56-2,06
TSC22D3	A_23_P217688	TSC22 domain family, member 3 (TSC22D3), transcript variant 2, mRNA [NM_004089]	97	1,426	1,47	1,44	1,91	0,55-3,56
A_24_P195528	A_24_P195528	Unknown	98	1,426	1,455	1,44	1,58	0,59-2,98
BAG3	A_23_P47077	BCL2-associated athanogene 3 (BAG3), mRNA [NM_004281]	99	1,426	1,44	1,44	1,82	0,44-3,50

Gene Name	Agilent ID	Description	SAM				Fold change	
			Called	FSN	FDR	q-val	mean	Range
PTPRE	A_24_P213503	protein tyrosine phosphatase, receptor type, E (PTPRE), transcript variant 1, mRNA [NM_006504]	100	2,139	2,139	1,678	0,58	0,10-1,94
VPRBP	A_23_P212595	Vpr (HIV-1) binding protein (VPRBP), mRNA [NM_014703]	101	2,139	2,118	1,55	1,47	1,03-3,20
FXR2	A_23_P4007	fragile X mental retardation, autosomal homolog 2 (FXR2), mRNA [NM_004860]	102	2,139	2,097	1,55	1,83	0,25-4,84
CYP2D6	A_23_P155123	cytochrome P450, family 2, subfamily D, polypeptide 6 (CYP2D6), transcript variant 1, mRNA [NM_000106]	103	2,139	2,077	1,55	1,51	0,46-3,38
A_24_P812172	A_24_P812172	Unknown	104	2,139	2,057	1,55	1,94	0,57-4,80
RASD1	A_23_P118392	RAS, dexamethasone-induced 1 (RASD1), mRNA [NM_016084]	105	2,139	2,037	1,55	3,14	0,88-21,55
C8orf73	A_23_P369641	chromosome 8 open reading frame 73 (C8orf73), mRNA [NM_001100878]	106	2,139	2,018	1,55	1,7	0,43-4,02
SLC13A2	A_23_P107307	solute carrier family 13 (sodium-dependent dicarboxylate transporter), member 2 (SLC13A2), mRNA [NM_003984]	107	2,139	1,999	1,55	1,62	0,87-2,71
HIRIP3	A_24_P142269	HIRA interacting protein 3 (HIRIP3), mRNA [NM_003609]	108	2,139	1,98	1,55	1,51	0,36-2,84
ENST00000307533	A_24_P89038	LOC93622 protein (Putative uncharacterized protein LOC93622) (cDNA, FLJ95718) [Source:UniProtKB/TrEMBL;Acc:Q8WU25] [ENST00000307533]	109	2,139	1,962	1,55	1,79	0,59-2,38
RGMB	A_24_P363100	RGM domain family, member B (RGMB), transcript variant 2, mRNA [NM_173670]	110	2,139	1,944	1,55	1,41	0,82-2,36
ARID5B	A_23_P97871	AT rich interactive domain 5B (MRF1-like) (ARID5B), mRNA [NM_032199]	111	2,139	1,927	1,55	2,11	0,81-3,00
IGF2BP2	A_23_P250156	insulin-like growth factor 2 mRNA binding protein 2 (IGF2BP2), transcript variant 1, mRNA [NM_006548]	112	2,139	1,91	1,55	1,6	1,09-3,09
SP100	A_24_P916816	nuclear autoantigen mRNA, partial cds; alternatively spliced, [L79989]	113	2,139	1,893	1,678	0,49	0,15-3,15
A_24_P349580	A_24_P349580	Unknown	114	2,139	1,876	1,55	1,38	0,75-2,38
A_24_P920081	A_24_P920081	Unknown	115	2,139	1,86	1,55	1,57	0,76-4,11
H6PD	A_24_P626850	hexose-6-phosphate dehydrogenase (glucose 1-dehydrogenase) (H6PD), mRNA [NM_004285]	116	2,139	1,844	1,55	2,6	0,26-4,61
SAPS2	A_23_P95353	SAPS domain family, member 2 (SAPS2), mRNA [NM_014678]	117	2,139	1,828	1,55	1,51	0,86-2,13
SPAG9	A_24_P365025	sperm associated antigen 9 (SPAG9), transcript variant 3, mRNA [NM_003971]	118	2,139	1,813	1,55	1,42	0,93-1,94
NDRG1	A_24_P38387	N-myc downstream regulated 1 (NDRG1), transcript variant 2, mRNA [NM_006096]	119	2,139	1,797	1,55	1,67	0,98-4,09
ENST00000312412	A_24_P690235	cDNA FLJ43844 fis, clone TESTI4006308, highly similar to Puromycin-sensitive aminopeptidase (EC 3,4,11,-), [AK125832]	120	2,139	1,782	1,55	1,49	0,14-2,16
A_24_P170365	A_24_P170365	Unknown	121	2,139	1,768	1,55	1,85	0,68-4,24
RHOU	A_23_P114814	ras homolog gene family, member U (RHOU), mRNA [NM_021205]	122	2,139	1,753	1,55	2,68	0,71-3,65
DHRS11	A_23_P27005	dehydrogenase/reductase (SDR family) member 11 (DHRS11), mRNA [NM_024308]	123	2,139	1,739	1,55	1,38	1,02-3,10

Gene Name	Agilent ID	Description	SAM				Fold change	
			Called	FSN	FDR	q-val	mean	Range
RNPEP	A_24_P68649	arginyl aminopeptidase (aminopeptidase B) (RNPEP), mRNA [NM_020216]	124	2,139	1,725	1,55	2,35	0,27-3,54
CHST10	A_23_P102351	carbohydrate sulfotransferase 10 (CHST10), mRNA [NM_004854]	125	2,139	1,711	1,55	1,5	0,33-3,35
CA314936	A_32_P140228	UI-CF-FN0-afi-d-17-0-UI,s1 UI-CF-FN0 Homo sapiens cDNA clone UI-CF-FN0-afi-d-17-0-UI 3', mRNA sequence [CA314936]	126	2,139	1,698	1,55	2,16	0,39-4,08
SSBP2	A_23_P33791	single-stranded DNA binding protein 2 (SSBP2), mRNA [NM_012446]	127	2,139	1,684	1,55	1,33	0,71-1,76
BRCC3	A_23_P217659	BRCA1/BRCA2-containing complex, subunit 3 (BRCC3), transcript variant 2, mRNA [NM_001018055]	128	2,139	1,671	1,55	1,47	0,89-3,17
SPG7	A_24_P5305	spastic paraplegia 7 (pure and complicated autosomal recessive) (SPG7), nuclear gene encoding mitochondrial protein, transcript variant 1, mRNA [NM_003119]	129	2,139	1,658	1,55	1,83	0,84-3,97
MFGE8	A_24_P133584	milk fat globule-EGF factor 8 protein (MFGE8), transcript variant 1, mRNA [NM_005928]	130	2,139	1,645	1,55	1,56	0,85-2,51
A_24_P152144	A_24_P152144	Unknown	131	2,139	1,633	1,55	1,51	0,63-2,53
CHKA	A_23_P136135	choline kinase alpha (CHKA), transcript variant 1, mRNA [NM_001277]	132	2,139	1,62	1,55	1,45	0,50-2,57
RBM8A	A_23_P305335	RNA binding motif protein 8A, mRNA (cDNA clone IMAGE:4687764), complete cds, [BC017770]	133	2,139	1,608	1,55	1,95	0,85-3,33
BC063542	A_32_P135790	cDNA clone IMAGE:4525305, **** WARNING: chimeric clone ****, [BC063542]	134	2,139	1,596	1,55	1,93	0,71-3,76
A_24_P460195	A_24_P460195	Unknown	135	2,139	1,584	1,55	2,36	0,37-5,36
DFNB31	A_23_P83351	deafness, autosomal recessive 31 (DFNB31), transcript variant 1, mRNA [NM_015404]	136	2,139	1,573	1,55	1,51	0,40-3,71
LOC388152	A_23_P305981	cDNA FLJ90297 fis, clone NT2RP2000447, moderately similar to GOLGIN-95, [AK074778]	137	2,139	1,561	1,55	1,68	0,84-2,65
CARS	A_24_P95439	cysteinyl-tRNA synthetase (CARS), transcript variant 4, mRNA [NM_001014438]	138	2,139	1,55	1,55	1,66	0,66-3,26
BI024548	A_32_P74942	BI024548 PM3-MT0205-300101-005-f07 MT0205 Homo sapiens cDNA, mRNA sequence [BI024548]	139	2,852	2,052	1,678	0,67	0,14-2,80
RASAL1	A_23_P139600	RAS protein activator like 1 (GAP1 like) (RASAL1), mRNA [NM_004658]	140	2,852	2,037	1,648	1,46	0,38-2,41
A_24_P33055	A_24_P33055	Unknown	141	2,852	2,023	1,648	1,38	0,92-2,47
CAMK1D	A_23_P124252	calcium/calmodulin-dependent protein kinase ID (CAMK1D), transcript variant 1, mRNA [NM_020397]	142	2,852	2,008	1,648	1,58	1,00-3,03
TMEM19	A_24_P358976	transmembrane protein 19 (TMEM19), mRNA [NM_018279]	143	2,852	1,994	1,678	0,71	0,40-0,93
ATP1A4	A_23_P160177	ATPase, Na+/K+ transporting, alpha 4 polypeptide (ATP1A4), transcript variant 1, mRNA [NM_144699]	144	2,852	1,98	1,648	1,64	0,77-3,35
ANO1	A_23_P98304	anoctamin 1, calcium activated chloride channel (ANO1), mRNA [NM_018043]	145	2,852	1,967	1,648	1,48	0,59-2,05
A_32_P78488	A_32_P78488	Unknown	146	2,852	1,953	1,648	1,35	0,90-1,96
UBL4A	A_23_P62361	ubiquitin-like 4A (UBL4A), mRNA [NM_014235]	147	2,852	1,94	1,648	1,64	0,36-3,54

Gene Name	Agilent ID	Description	SAM				Fold change	
			Called	FSN	FDR	q-val	mean	Range
SDHA	A_32_P67259	succinate dehydrogenase complex, subunit A, flavoprotein (Fp) (SDHA), nuclear gene encoding mitochondrial protein, mRNA [NM_004168]	148	2,852	1,927	1,648	1,42	0,54-2,68
CNOT3	A_23_P38987	CCR4-NOT transcription complex, subunit 3 (CNOT3), mRNA [NM_014516]	149	2,852	1,914	1,648	1,3	0,75-2,22
ARHGEF9	A_24_P254551	Cdc42 guanine nucleotide exchange factor (GEF) 9 (ARHGEF9), mRNA [NM_015185]	150	2,852	1,901	1,648	1,8	0,76-4,15
STK35	A_24_P940537	serine/threonine kinase 35 (STK35), mRNA [NM_080836]	151	2,852	1,889	1,648	1,6	0,52-3,00
DDR1	A_23_P93311	discoidin domain receptor tyrosine kinase 1 (DDR1), transcript variant 1, mRNA [NM_013993]	152	2,852	1,876	1,648	1,74	0,40-3,20
C3orf39	A_24_P217330	chromosome 3 open reading frame 39 (C3orf39), mRNA [NM_032806]	153	2,852	1,864	1,648	1,42	0,48-2,69
ZKSCAN5	A_24_P317637	zinc finger with KRAB and SCAN domains 5 (ZKSCAN5), transcript variant 1, mRNA [NM_014569]	154	2,852	1,852	1,648	1,61	0,73-2,22
TBC1D17	A_23_P130731	TBC1 domain family, member 17 (TBC1D17), mRNA [NM_024682]	155	2,852	1,84	1,648	1,26	0,36-2,11
RNF215	A_32_P420563	ring finger protein 215 (RNF215), mRNA [NM_001017981]	156	2,852	1,828	1,648	1,79	0,61-2,98
LOC100130713	A_24_P119405	cDNA FLJ39247 fis, clone OCBBF2008520, [AK096566]	157	2,852	1,816	1,648	1,81	0,96-3,49
PPP2R3B	A_23_P45517	protein phosphatase 2 (formerly 2A), regulatory subunit B'', beta (PPP2R3B), transcript variant 1, mRNA [NM_013239]	158	2,852	1,805	1,648	1,29	0,54-2,17
EPHA1	A_23_P157333	EPH receptor A1 (EPHA1), mRNA [NM_005232]	159	2,852	1,794	1,648	1,67	0,55-2,95
GPR65	A_23_P14564	G protein-coupled receptor 65 (GPR65), mRNA [NM_003608]	160	2,852	1,782	1,678	0,61	0,35-4,64
RMND5A	A_24_P114339	required for meiotic nuclear division 5 homolog A (S, cerevisiae) (RMND5A), mRNA [NM_022780]	161	2,852	1,771	1,648	2,08	0,76-4,92
CR616003	A_24_P65597	gb|full-length cDNA clone CS0DM012YB14 of Fetal liver of Homo sapiens (human), [CR616003]	162	2,852	1,76	1,648	2,41	0,21-15,71
CYR61	A_23_P46426	cysteine-rich, angiogenic inducer, 61 (CYR61), mRNA [NM_001554]	163	2,852	1,75	1,648	4,07	0,61-12,88
ANGEL2	A_24_P28622	angel homolog 2 (Drosophila) (ANGEL2), mRNA [NM_144567]	164	2,852	1,739	1,678	0,79	0,54-0,98
RNF185	A_32_P15421	ring finger protein 185 (RNF185), transcript variant 1, mRNA [NM_152267]	165	2,852	1,728	1,648	1,5	0,62-2,03
UBE2MP1	A_24_P239017	ubiquitin-conjugating enzyme E2M pseudogene 1 (UBE2MP1), non-coding RNA [NR_002837]	166	2,852	1,718	1,648	1,31	0,42-9,42
TUBA4A	A_23_P102109	tubulin, alpha 4a (TUBA4A), mRNA [NM_006000]	167	2,852	1,708	1,648	1,46	0,39-3,22
TSPAN12	A_23_P145984	tetraspanin 12 (TSPAN12), mRNA [NM_012338]	168	2,852	1,698	1,648	1,64	1,04-3,43
DDR1	A_24_P367289	discoidin domain receptor tyrosine kinase 1 (DDR1), transcript variant 1, mRNA [NM_013993]	169	2,852	1,687	1,648	1,51	0,29-6,52
X05126	A_32_P64936	Human fibroblast mRNA fragment with Alu sequence (pRHF11), [X05126]	170	2,852	1,678	1,678	0,66	0,39-1,43
GLTPD2	A_24_P392925	glycolipid transfer protein domain containing 2 (GLTPD2), mRNA [NM_001014985]	171	2,852	1,668	1,648	1,57	0,77-3,48

Gene Name	Agilent ID	Description	SAM				Fold change	
			Called	FSN	FDR	q-val	mean	Range
SLC22A12	A_23_P35809	solute carrier family 22 (organic anion/urate transporter), member 12 (SLC22A12), transcript variant 1, mRNA [NM_144585]	172	2,852	1,658	1,648	1,68	0,91-3,22
SIX5	A_23_P328687	SIX homeobox 5 (SIX5), mRNA [NM_175875]	173	2,852	1,648	1,648	1,43	0,60-2,08
IMMT	A_24_P351304	inner membrane protein, mitochondrial (mitofilin) (IMMT), nuclear gene encoding mitochondrial protein, transcript variant 1, mRNA [NM_006839]	174	3,565	2,049	1,791	1,23	0,59-1,54
ZNF628	A_32_P409222	zinc finger protein 628 (ZNF628), mRNA [NM_033113]	175	3,565	2,037	1,791	1,25	0,63-1,73
L3MBTL	A_23_P501961	l(3)mbt-like (Drosophila) (L3MBTL), transcript variant II, mRNA [NM_032107]	176	3,565	2,025	1,791	1,63	0,85-2,52
DGCR2	A_24_P125881	DiGeorge syndrome critical region gene 2 (DGCR2), mRNA [NM_005137]	177	3,565	2,014	1,791	1,31	0,58-1,59
WAPAL	A_23_P342185	wings apart-like homolog (Drosophila) (WAPAL), mRNA [NM_015045]	178	3,565	2,003	1,791	1,31	0,87-1,91
B4GALT4	A_32_P103945	UDP-Gal:betaGlcNAc beta 1,4-galactosyltransferase, polypeptide 4 (B4GALT4), transcript variant 1, mRNA [NM_212543]	179	3,565	1,992	1,791	1,45	0,82-2,35
SPRY1	A_23_P144476	sprouty homolog 1, antagonist of FGF signaling (Drosophila) (SPRY1), transcript variant 2, mRNA [NM_199327]	180	3,565	1,98	1,791	1,69	0,77-8,90
LRP5L	A_24_P387514	low density lipoprotein receptor-related protein 5-like (LRP5L), transcript variant 1, mRNA [NM_182492]	181	3,565	1,97	1,791	1,56	0,98-3,46
ARHGEF5	A_24_P389218	Rho guanine nucleotide exchange factor (GEF) 5 (ARHGEF5), mRNA [NM_005435]	182	3,565	1,959	1,791	1,9	0,43-4,10
NFRKB	A_23_P24485	nuclear factor related to kappaB binding protein (NFRKB), transcript variant 2, mRNA [NM_006165]	183	3,565	1,948	1,791	1,61	0,63-3,25
RHOV	A_23_P117912	Rho-related GTP-binding protein RhoV (Wnt-1 responsive Cdc42 homolog 2)(WRCH-2)(CDC42-like GTPase 2)(GTP-binding protein-like 2)(Rho GTPase-like protein ARHV) [Source:UniProtKB/Swiss-Prot;Acc:Q96L33] [ENST00000220507]	184	3,565	1,937	1,791	1,6	0,25-5,45
CCDC52	A_23_P109733	coiled-coil domain containing 52 (CCDC52), mRNA [NM_144718]	185	3,565	1,927	1,791	1,47	0,52-1,90
SLC25A27	A_23_P81721	solute carrier family 25, member 27 (SLC25A27), nuclear gene encoding mitochondrial protein, mRNA [NM_004277]	186	3,565	1,917	1,791	1,82	0,86-2,93
SCRN2	A_23_P4323	secernin 2 (SCRN2), mRNA [NM_138355]	187	3,565	1,906	1,791	1,35	0,82-2,17
AK022337	A_24_P825969	cDNA FLJ12275 fis, clone MAMMA1001686, [AK022337]	188	3,565	1,896	1,791	1,6	0,95-3,98
C20orf151	A_32_P331052	chromosome 20 open reading frame 151 (C20orf151), mRNA [NM_080833]	189	3,565	1,886	1,791	1,32	0,38-2,32
GABARAP	A_23_P27075	GABA(A) receptor-associated protein (GABARAP), mRNA [NM_007278]	190	3,565	1,876	1,81	0,79	0,57-1,43
CYTH2	A_23_P119377	cytohesin 2 (CYTH2), transcript variant 2, mRNA [NM_004228]	191	3,565	1,866	1,791	1,58	0,48-4,52
THC2564025	A_24_P936051	BC065015 transmembrane protein 4 {Homo sapiens} (exp=-1; wgp=0; cg=0), partial (8%) [THC2564025]	192	3,565	1,857	1,791	1,42	0,79-2,76
SETD1A	A_23_P129678	SET domain containing 1A (SETD1A), mRNA [NM_014712]	193	3,565	1,847	1,791	1,44	0,32-2,89

Gene Name	Agilent ID	Description	SAM				Fold change	
			Called	FSN	FDR	q-val	mean	Range
EXDL2	A_23_P205296	exonuclease 3'-5' domain-like 2 (EXDL2), mRNA [NM_018199]	194	3,565	1,838	1,791	1,48	0,57-2,63
EID2	A_23_P365844	EP300 interacting inhibitor of differentiation 2 (EID2), mRNA [NM_153232]	195	3,565	1,828	1,791	1,3	0,73-2,81
CYR61	A_24_P370946	cysteine-rich, angiogenic inducer, 61 (CYR61), mRNA [NM_001554]	196	3,565	1,819	1,791	5,51	0,36-22,80
RFT1	A_24_P93741	RFT1 homolog (S, cerevisiae) (RFT1), mRNA [NM_052859]	197	3,565	1,81	1,81	0,64	0,49-1,18
BAHCC1	A_23_P49539	BAH domain and coiled-coil containing 1 (BAHCC1), mRNA [NM_001080519]	198	3,565	1,8	1,791	1,55	0,74-5,59
RIPK4	A_23_P211267	receptor-interacting serine-threonine kinase 4 (RIPK4), mRNA [NM_020639]	199	3,565	1,791	1,791	2,08	0,64-4,59
HGS	A_24_P125993	hepatocyte growth factor-regulated tyrosine kinase substrate (HGS), mRNA [NM_004712]	200	4,278	2,139	2,008	1,76	0,59-2,60
A_24_P392099	A_24_P392099	Unknown	201	4,278	2,128	2,008	1,44	0,38-3,60
A_24_P455100	A_24_P455100	Unknown	202	4,278	2,118	2,008	1,38	1,02-2,06
FOSB	A_23_P429998	FBJ murine osteosarcoma viral oncogene homolog B (FOSB), transcript variant 1, mRNA [NM_006732]	203	4,278	2,107	2,008	2,79	0,96-24,26
DCAKD	A_24_P58331	dephospho-CoA kinase domain containing (DCAKD), transcript variant 1, mRNA [NM_024819]	204	4,278	2,097	2,008	1,89	0,41-4,80
SUV420H2	A_23_P430044	suppressor of variegation 4-20 homolog 2 (Drosophila) (SUV420H2), mRNA [NM_032701]	205	4,278	2,087	2,008	1,41	0,89-2,29
EWSR1	A_23_P91657	Ewing sarcoma breakpoint region 1 (EWSR1), transcript variant EWS-b, mRNA [NM_013986]	206	4,278	2,077	2,008	1,57	0,38-2,43
OSMR	A_24_P145134	oncostatin M receptor, mRNA (cDNA clone IMAGE:4043935), complete cds, [BC010943]	207	4,278	2,067	2,008	1,32	0,54-2,17
SFPQ	A_24_P105283	splicing factor proline/glutamine-rich (polypyrimidine tract binding protein associated) (SFPQ), mRNA [NM_005066]	208	4,278	2,057	2,008	1,82	0,64-3,83
BCKDHB	A_24_P914513	branched chain keto acid dehydrogenase E1, beta polypeptide (BCKDHB), nuclear gene encoding mitochondrial protein, transcript variant 1, mRNA [NM_183050]	209	4,278	2,047	2,008	1,55	0,62-2,08
SEC24A	A_23_P329593	SEC24 related gene family, member A (S, cerevisiae), mRNA (cDNA clone MGC:12985 IMAGE:3355949), complete cds, [BC019341]	210	4,278	2,037	2,008	1,69	0,95-4,92
SPRY4	A_24_P269062	sprouty homolog 4 (Drosophila) (SPRY4), transcript variant 1, mRNA [NM_030964]	211	4,278	2,027	2,008	1,47	0,61-2,20
A_24_P837537	A_24_P837537	Unknown	212	4,278	2,018	2,008	1,67	0,81-3,35
PC	A_23_P161647	pyruvate carboxylase (PC), nuclear gene encoding mitochondrial protein, transcript variant 3, mRNA [NM_001040716]	213	4,278	2,008	2,008	2,22	0,72-9,75
THC2662244	A_32_P108033	Q26SG8_XANP2 (Q26SG8) Cytochrome C-type biogenesis protein CycL precursor, partial (8%) [THC2662244]	214	4,278	1,999	1,999	0,48	0,22-2,06
ACTR3B	A_23_P123193	ARP3 actin-related protein 3 homolog B (yeast) (ACTR3B), transcript variant 1, mRNA [NM_020445]	215	4,991	2,321	2,199	1,54	0,79-2,04
RUNDC2B	A_32_P66020	RUN domain containing 2B (RUNDC2B), mRNA [NM_001012391]	216	4,991	2,311	2,189	0,74	0,40-1,69

Gene Name	Agilent ID	Description	SAM				Fold change	
			Called	FSN	FDR	q-val	mean	Range
DUSP1	A_23_P110712	dual specificity phosphatase 1 (DUSP1), mRNA [NM_004417]	217	4,991	2,3	2,199	3,18	0,49-12,69
EXDL2	A_23_P205293	exonuclease 3'-5' domain-like 2 (EXDL2), mRNA [NM_018199]	218	4,991	2,289	2,199	1,31	0,58-2,17
KIAA0922	A_23_P257250	KIAA0922 (KIAA0922), transcript variant 2, mRNA [NM_015196]	219	4,991	2,279	2,199	1,75	0,83-6,14
ADAMDEC1	A_23_P256425	ADAM-like, decysin 1 (ADAMDEC1), mRNA [NM_014479]	220	4,991	2,269	2,189	0,59	0,11-6,10
LOC644450	A_24_P910030	hypothetical protein LOC644450, mRNA (cDNA clone IMAGE:4606942), partial cds, [BC022881]	221	4,991	2,258	2,189	0,63	0,41-1,24
ZFP36	A_23_P39237	zinc finger protein 36, C3H type, homolog (mouse) (ZFP36), mRNA [NM_003407]	222	4,991	2,248	2,199	2,21	0,07-23,35
PIGS	A_23_P66715	phosphatidylinositol glycan anchor biosynthesis, class S (PIGS), mRNA [NM_033198]	223	4,991	2,238	2,199	1,45	0,45-2,59
TRAM2	A_23_P58937	translocation associated membrane protein 2 (TRAM2), mRNA [NM_012288]	224	4,991	2,228	2,189	0,7	0,36-1,18
CTNNB1	A_23_P29499	catenin (cadherin-associated protein), beta 1, 88kDa (CTNNB1), mRNA [NM_001904]	225	4,991	2,218	2,199	1,63	0,79-5,30
PEX5	A_24_P372932	peroxisomal biogenesis factor 5 (PEX5), transcript variant 2, mRNA [NM_000319]	226	4,991	2,208	2,199	1,7	0,56-5,88
TH1L	A_24_P222126	TH1-like (Drosophila) (TH1L), transcript variant 1, mRNA [NM_198976]	227	4,991	2,199	2,199	1,3	0,47-2,10
THC2498220	A_24_P936252	thc|Q30VC0_DESDG (Q30VC0) Flagellar biosynthetic protein FliP, partial (8%) [THC2498220]	228	4,991	2,189	2,189	0,43	0,11-1,25
A_32_P71864	A_32_P71864	Unknown	229	5,704	2,491	2,427	1,28	0,96-2,05
RAVER2	A_23_P328323	ribonucleoprotein, PTB-binding 2 (RAVER2), mRNA [NM_018211]	230	5,704	2,48	2,427	1,66	0,99-3,04
CSTF2T	A_23_P12730	cleavage stimulation factor, 3' pre-RNA, subunit 2, 64kDa, tau variant (CSTF2T), mRNA [NM_015235]	231	5,704	2,469	2,437	0,62	0,39-1,09
FAM116B	A_24_P659036	family with sequence similarity 116, member B (FAM116B), mRNA [NM_001001794]	232	5,704	2,458	2,427	1,42	0,28-2,78
FLJ22795	A_23_P320478	similar to cis-Golgi matrix protein GM130, mRNA (cDNA clone MGC:70902 IMAGE:6144741), complete cds, [BC065260]	233	5,704	2,448	2,427	1,78	0,74-3,57
EVI2B	A_23_P66694	ecotropic viral integration site 2B (EVI2B), mRNA [NM_006495]	234	5,704	2,437	2,437	0,56	0,15-3,17
INO80	A_24_P39454	INO80 homolog (S, cerevisiae) (INO80), mRNA [NM_017553]	235	5,704	2,427	2,427	1,51	0,63-2,66

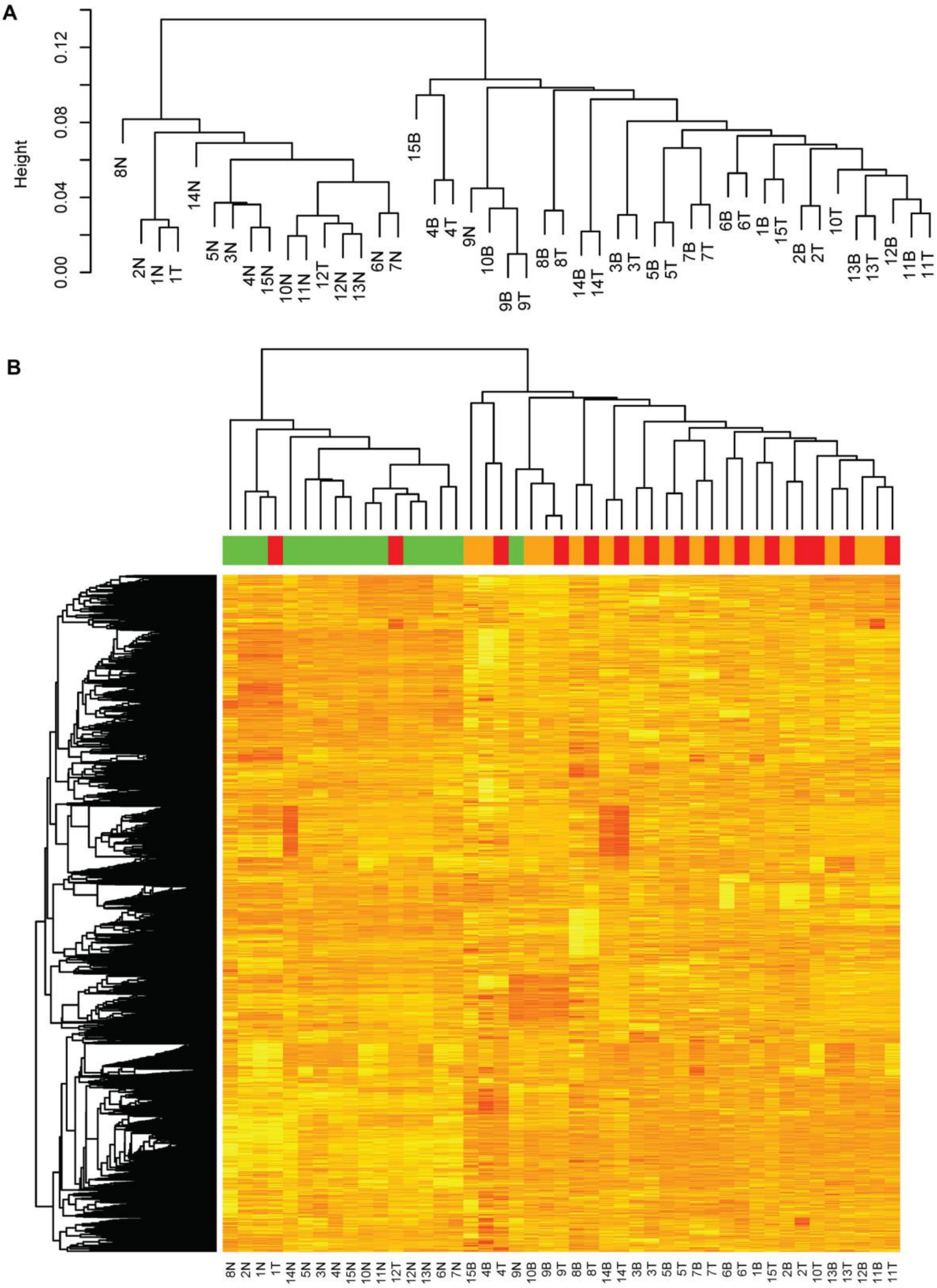

Supplementary fig 1

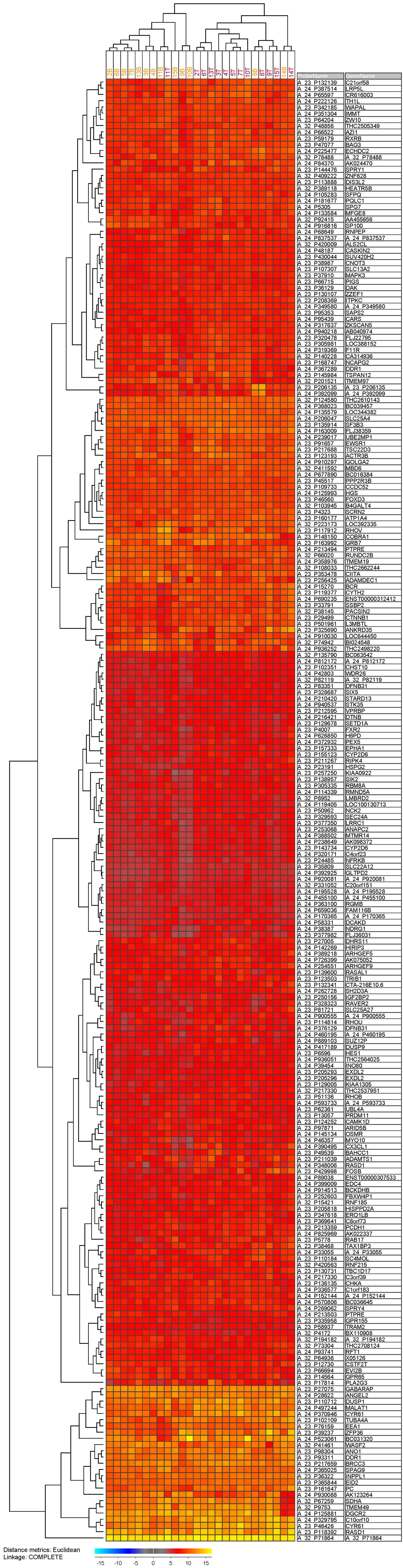
2B
6B
5B
7B
13B
3B
4B
11B
11T
15B
9B
10B
2T
6T
12T
13T
3T
4T
5T
7T
10T
8B
8T
9T
15T
14B
14T
ProbeName
GeneName
Distance metrics: Euclidean
Linkage: COMPLETE
-15
-10
-5
0
5
10
15

Supplementary fig 2

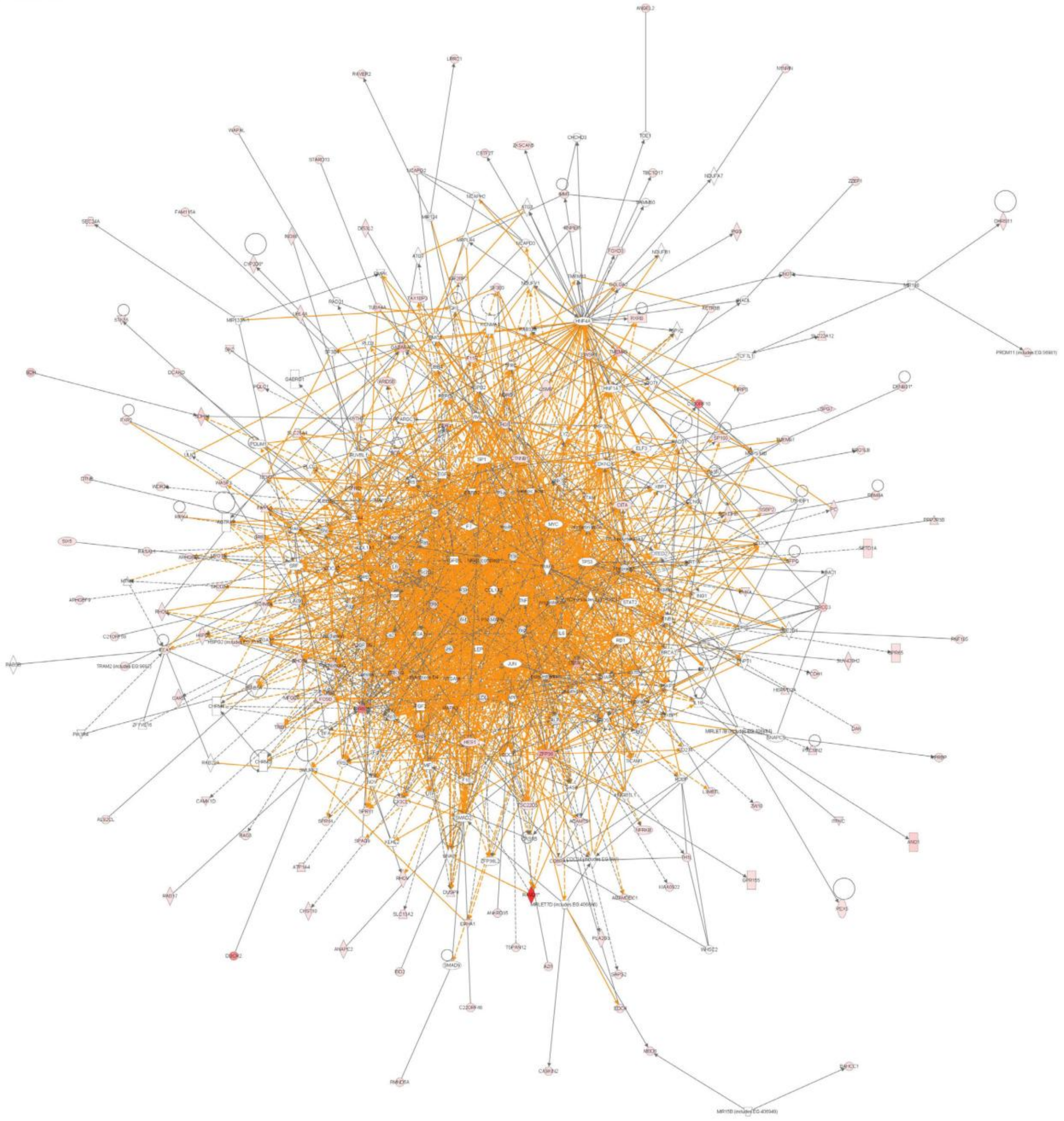

Supplementary fig 3
